# Reproductive responses of birds to experimental food supplementation: a meta-analysis

**DOI:** 10.1186/s12983-014-0080-y

**Published:** 2014-10-31

**Authors:** Lise Ruffino, Pälvi Salo, Elina Koivisto, Peter B Banks, Erkki Korpimäki

**Affiliations:** Section of Ecology, Department of Biology, University of Turku, FI-20014 Turku, Finland; School of Biological Sciences, The University of Sydney, Sydney, Australia

**Keywords:** Effect size, Feeding experiment, Population regulation, Reproductive performance, Resource competition, Wildlife management

## Abstract

**Introduction:**

Food availability is an important environmental cue for animals for deciding how much to invest in reproduction, and it ultimately affects population size. The importance of food limitation has been extensively studied in terrestrial vertebrate populations, especially in birds, by experimentally manipulating food supply. However, the factors explaining variation in reproductive decisions in response to food supplementation remain unclear. By performing meta-analyses, we aim to quantify the extent to which supplementary feeding affects several reproductive parameters in birds, and identify the key factors (life-history traits, behavioural factors, environmental factors, and experimental design) that can induce variation in laying date, clutch size and breeding success (i.e., number of fledglings produced) in response to food supplementation.

**Results:**

Food supplementation produced variable but mostly positive effects across reproductive parameters in a total of 201 experiments from 82 independent studies. The outcomes of the food effect were modulated by environmental factors, e.g., laying dates advanced more towards low latitudes, and food supplementation appeared not to produce any obvious effect on bird reproduction when the background level of food abundance in the environment was high. Moreover, the increase in clutch size following food addition was more pronounced in birds that cache food, as compared to birds that do not. Supplementation timing was identified as a major cause of variation in breeding success responses. We also document the absence of a detectable food effect on clutch size and breeding success when the target species had poor access to the feed due to competitive interactions with other animals.

**Conclusions:**

Our findings indicate that, from the pool of bird species and environments reviewed, extra food is allocated to immediate reproduction in most cases. Our results also support the view that bird species have evolved different life-history strategies to cope with environmental variability in food supply. However, we encourage more research at low latitudes to gain knowledge on how resource allocation in birds changes along a latitudinal gradient. Our results also emphasize the importance of developing experimental designs that minimise competition for the supplemented food and the risk of reproductive bottle-necks due to inappropriate supplementation timings.

**Electronic supplementary material:**

The online version of this article (doi:10.1186/s12983-014-0080-y) contains supplementary material, which is available to authorized users.

## Introduction

Resource availability is an important determinant of the demography and distribution of many species, and it also affects energy allocation strategies in individuals. The energy involved to obtain food and process it is tightly related to the physiology and behaviour of animals, and the proportion of total energy and nutrients that is allocated to reproduction (i.e., reproductive effort) causes variation in life histories across individuals [1]. The importance of food limitation in nature continues to puzzle ecologists, as it can vary in time and across species, breeding stages and environments. Food limitation is likely less important in systems where predation or critical resources other than food keeps populations below their carrying capacity (e.g., [2]). Conversely, food limitation can be inferred when a shortage of food or any critical nutrients results in lower reproductive performance and/or reduced survival [1,3,4], that ultimately leads to a decline in growth rate of the focal population. Food limitation can have short (e.g., reproductive effort) and long-term (e.g., adult survival and future reproduction) life-history consequences. Variation in food availability in space and time is therefore an important cue for animals to adjust their reproductive decisions: it can provide information on where and when to breed, with whom to breed, and how much to invest in offspring (e.g., [1,5,6]).

Birds have proven to be useful models in exploring the ecological and biological factors influencing the level of reproductive investment. Some evidence for the effects of food supply on the reproduction of bird populations comes from correlations where natural spatial or temporal variations in food availability have been linked to observed changes in population growth. For instance, the breeding success of specialised avian predators is closely correlated with the marked population fluctuations of their main prey, i.e., small rodents (e.g., [7,8]). Moreover, heavy rainfalls in arid systems positively correlate with increases in bird fecundity, presumably via enhanced food supply (e.g., [9]). Other examples of resource pulse events (i.e., sudden large increases in food supply) that have triggered a reproductive response in birds include insect outbreaks [10] and seed mast events [11].

While such correlations provide apparent evidence about the extent of food limitation in bird reproduction, they do not show causality. In addition, they are, in a sense, special cases as resource pulse events and prey population cycles generate highly contrasted levels of resource abundance, as opposed to more subtle differences in food availability that may be more difficult to identify. On the other hand, supplementary feeding of birds, which involves provisioning with types of foods in quantities that would not be available naturally, has proven to be a useful alternative method to quantitatively assess the importance of food supply in the reproductive decisions of wild bird populations, by taking into account potential confounding factors. In cases where food abundance in nature is used by birds as a cue for food conditions, manipulating food supply can experimentally address how environmental variation (in food supply) shapes life history [1].

The effects of food supplementation on various components of bird reproduction have been experimentally studied in a variety of taxa and environments, and on various components of reproduction. However, the results have been somewhat equivocal: while most studies have reported advancements of laying dates, increases in chick growth rate and breeding success [12], others have found no clear fitness benefits, e.g. no obvious effect on clutch size [13,14], or have even described negative effects on clutch size and breeding success [15,16]. The reasons for inconsistent results are not well known but may relate to the life-history traits of the species, the environmental conditions or the experimental design. The level of intra- and inter-specific competition for food, which can depend on the efficiency of the food delivery and the capacity of the recipient species to defend the food, might also explain the variable responses of birds to supplemented food [17].

Reports on the effects of supplemental food on bird reproduction have primarily been qualitative or based on vote-counting [1,4,12,18,19], thus general predictions about the effects of food supply on different components of reproduction across bird taxa and environments have not been tested quantitatively (but see [20]). In addition, recent quantitative analyses on the relative effects of latitude, broodedness and migratory status on the advancement of laying date in response to food supplementation have revealed contrasting results, leading to some debate [20-22]. In this paper we present a meta-analysis that synthesises the outcomes of food supplementation experiments on several bird reproductive parameters across 48 species. We specifically aim to (i) quantify the extent to which supplementary feeding induces an advancement of egg-laying and an increase in other components of the bird breeding cycle, i.e. clutch size, egg size, hatching success, brood size, chick body mass and breeding success (i.e., number of fledglings produced), and (ii) identify the key factors (including life-history traits, behavioural factors, environmental factors, experimental design) that can induce variation in laying date, clutch size and breeding success in response to food supplementation. These results will provide key-elements for a better understanding of the impact of food limitation on wild bird populations, and they can have important implications for the conservation of endangered populations whose long-term persistence might require food provisioning by practitioners.

## Results

In total, we retained 82 independent studies (201 experiments) that tested the effect of food supplementation on bird reproduction. These experiments were carried out primarily on birds of prey (19 studies), corvids (11 studies) and other passerines (38 studies), with only 15 experiments on seabirds (7 studies), eight on wetland birds (5 studies) and two on other bird families (i.e., Alcedinidae, Phasianidae). Studies were biased towards northern latitudes (≥50°N; n = 44 studies), with only 10% (n = 8) of studies at latitudes below 30°N or 30°S. Forty one studies (50%) tested the effect of food supplementation over at least two years.

**Figure 1:**
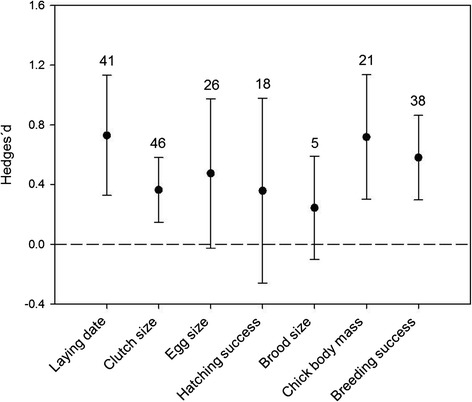
**Overall effects of food supplementation on reproductive parameters of birds.** Mean effect sizes (Hedges´*d*) and 95% confidence intervals are presented, with sample size (number of experiments) above bars.

The effect of food supplementation was not consistent across all reproductive parameters. The 95% confidence intervals indicated that the mean effect size of food supplementation was significantly positive for four reproductive parameters: laying date, clutch size, chick body mass and breeding success (Figure 1). Conversely, food supplementation had no detectable effect on egg size, hatching success or brood size (Figure 1).

**Figure 2:**
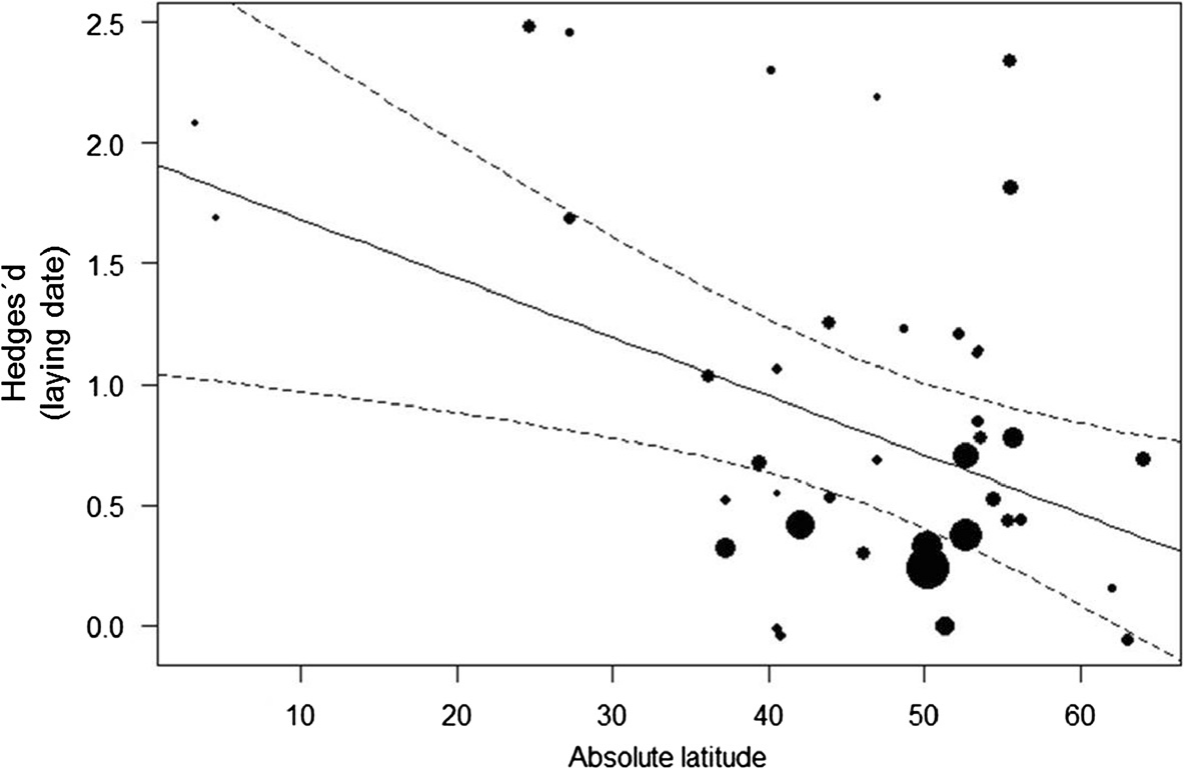
**Effect of latitude on advancement of laying date.** Effect of latitude of the study location on the degree of advancement of egg-laying date in response to food supplementation. Each dot represents one experiment and larger dots represent lower within-study variance, which has been used as a weighing factor in the analysis. Slope of the meta-regression = −0.024 (SE = 0.009). The regression line is bounded by 95% confidence intervals. Sample size is 41 experiments.

The amplitude of laying date advancement was mainly explained by the latitude of the study area (Tables 1, 2), whereby egg-laying was most advanced at lower latitudes (Figure 2). By comparison, migratory status, elevation of the study area, broodedness and diet received no support for explaining variation in laying date (Table 2). Despite the moderate AICc weighting of accessibility to the feed, this variable did not significantly explained variation in laying date (*Q* = 2.02; *P* = 0.36; see also Additional file 1).

**Table 1:** **Performance of models explaining variation in laying date, clutch size and breeding success of birds**

**Model rank**	**Model**	**log-likelihood**	**AICc**	**ΔAICc**	***wi***
Laying date					
1	rma.mv(d ~ Latitude + FoodAccess)	−37.02	88.84	0.00	0.13
2	rma.mv(d ~ Latitude)	−40.44	90.06	1.22	0.07
3	rma.mv(d ~ Latitude + FoodAccess + Elevation)	−36.10	90.20	1.36	0.07
4	rma.mv(d ~ Latitude + FoodAccess + Migratory + Broodedness)	−36.13	90.26	1.42	0.07
5	rma.mv(d ~ Latitude + FoodAccess + Migratory)	−36.13	90.26	1.42	0.07
6	rma.mv(d ~ Latitude + Elevation)	−39.35	90.58	1.74	0.06
7	rma.mv(d ~ Latitude + FoodAccess + Diet)	−36.48	90.97	2.13	0.05
8	rma.mv(d ~ Latitude + Migratory)	−39.64	91.16	2.32	0.04
9	rma.mv(d ~ Latitude + Diet)	−39.73	91.34	2.50	0.04
10	rma.mv(d ~ Latitude + FoodAccess + Broodedness)	−36.70	91.39	2.55	0.04
30	rma.mv(d ~ 1)	−57.93	122.53	7.08	0.00
Clutch size					
1	rma.mv(d ~ FoodAccess + FoodCaching)	−26.77	67.94	0.00	0.13
2	rma.mv(d ~ FoodAccess + FoodCaching + MaxClutchSize)	−25.72	68.84	0.90	0.08
3	rma.mv(d ~ FoodCaching + MaxClutchSize)	−28.68	68.98	1.04	0.08
4	rma.mv(d ~ FoodAccess)	−28.75	69.13	1.19	0.07
5	rma.mv(d ~ FoodAccess + FoodCaching + Latitude)	−26.32	70.04	2.10	0.04
6	rma.mv(d ~ FoodAccess + Latitude)	−27.97	70.35	2.41	0.04
7	rma.mv(d ~ FoodAccess + FoodCaching + MaxClutchSize + Latitude)	−24.86	70.37	2.43	0.04
8	rma.mv(d ~ FoodAccess + FoodCaching + Mass)	−26.58	70.56	2.62	0.03
9	rma.mv(d ~ FoodCaching)	−30.78	70.58	2.64	0.03
10	rma.mv(d ~ FoodAccess + MaxClutchSize)	−28.10	70.61	2.67	0.03
50	rma.mv(d ~ 1)	−50.21	107.01	11.52	0.00
Breeding success					
1	rma.mv(d ~ Timing + FoodAccess)	−39.21	93.53	0.00	0.13
2	rma.mv(d ~ Timing)	−42.14	93.57	0.04	0.12
3	rma.mv(d ~ Timing + Diet)	−40.81	93.69	0.17	0.12
4	rma.mv(d ~ Timing + FoodAccess + Diet)	−37.86	94.19	0.66	0.09
5	rma.mv(d ~ Timing + FoodCaching)	−41.40	94.86	1.33	0.06
6	rma.mv(d ~ Timing + Mass)	−41.49	95.04	1.52	0.06
7	rma.mv(d ~ Timing + FoodAccess + FoodCaching)	−38.30	95.09	1.56	0.06
8	rma.mv(d ~ Timing + FoodCaching + Diet)	−40.04	95.19	1.67	0.05
9	rma.mv(d ~ Timing + Diet + Mass)	−40.18	95.47	1.94	0.05
10	rma.mv(d ~ Timing + FoodAccess + Mass)	−38.60	95.68	2.15	0.04
25	rma.mv(d ~ 1)	−53.35	113.44	20.08	0.00

Only the 10 best models and the null models are shown.

**Table 2:** **Variable weights from model selection analyses**

**Model type**	**Variables**	**Variable weight from AICc**
Laying date		
	Latitude	0.92
	Food accessibility	0.54
	Migratory status	0.36
	Elevation	0.31
	Broodedness	0.28
	Diet	0.23
Clutch size		
	Food accessibility	0.74
	Food caching	0.62
	Maximal clutch size	0.42
	Latitude	0.29
	Bodymass	0.22
	Diet	0.21
Breeding success		
	Timing	0.92
	Food accessibility	0.47
	Diet	0.44
	Food caching	0.32
	Body mass	0.20

Variable weights were calculated as the sum of AICc weights of all candidate models including a given variable.

**Figure 3:**
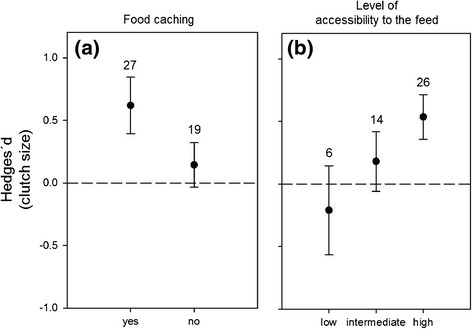
**Effects of food caching and food accessibility on variation in clutch size.** Effect of **(a)** food caching and **(b)** the level of accessibility to the feed on variation in clutch size in response to food supplementation in birds. Mean effect sizes (Hedges´*d*) and 95% confidence intervals are presented, with sample size (number of experiments) above bars.

Accessibility to the feed and food caching behaviour received strong support as predictors of clutch size variation (Tables 1, 2). There was no detectable effect of food supplementation on clutch size when the level of food accessibility was deemed low or intermediate, or in birds that do not cache food (Figure 3). Conversely, birds that cache and had full access to the food increased their clutch size in response to food supplementation (Figure 3). Maximal clutch size, latitude, body mass and diet all received low support as predictors of clutch size variation (Table 2; see also Additional file 1).

**Figure 4:**
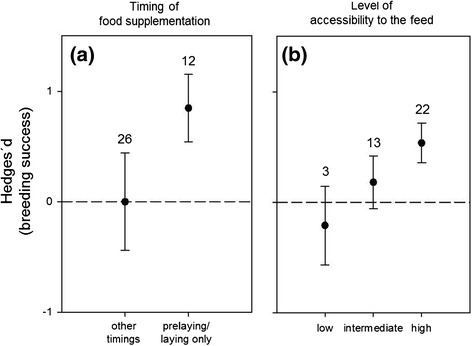
**Effects of timing and food accessibility on variation in breeding success.** Effect of **(a)** timing of food supplementation and **(b)** level of accessibility to the feed on breeding success variation in response to food supplementation in birds. Mean effect sizes (Hedges´*d*) and 95% confidence intervals are presented, with sample size (number of experiments) above bars.

Timing of food supplementation appeared as a strong predictor of the variation in breeding success, i.e. number of fledglings produced (Tables 1, 2). Studies that provided food only before and/or during egg-laying produced no detectable effect, whereas positive effect sizes were recorded for studies where food was either added from hatching to fledging or throughout the breeding season (Figure 4). Accessibility to the feed had a moderate AICc weight (0.47) in the series of models tested. Further inspection of the graphical results indicated that when the level of food accessibility was low or intermediate, food supplementation had on average no effect on breeding success, as opposed to when the level of accessibility to the food was high (*Q* = 9.59; *P* = 0.02; Figure 4). Diet, food caching or body mass did not explain variation in breeding success following food supplementation (Table 2; see also Additional file 1).

**Figure 5:**
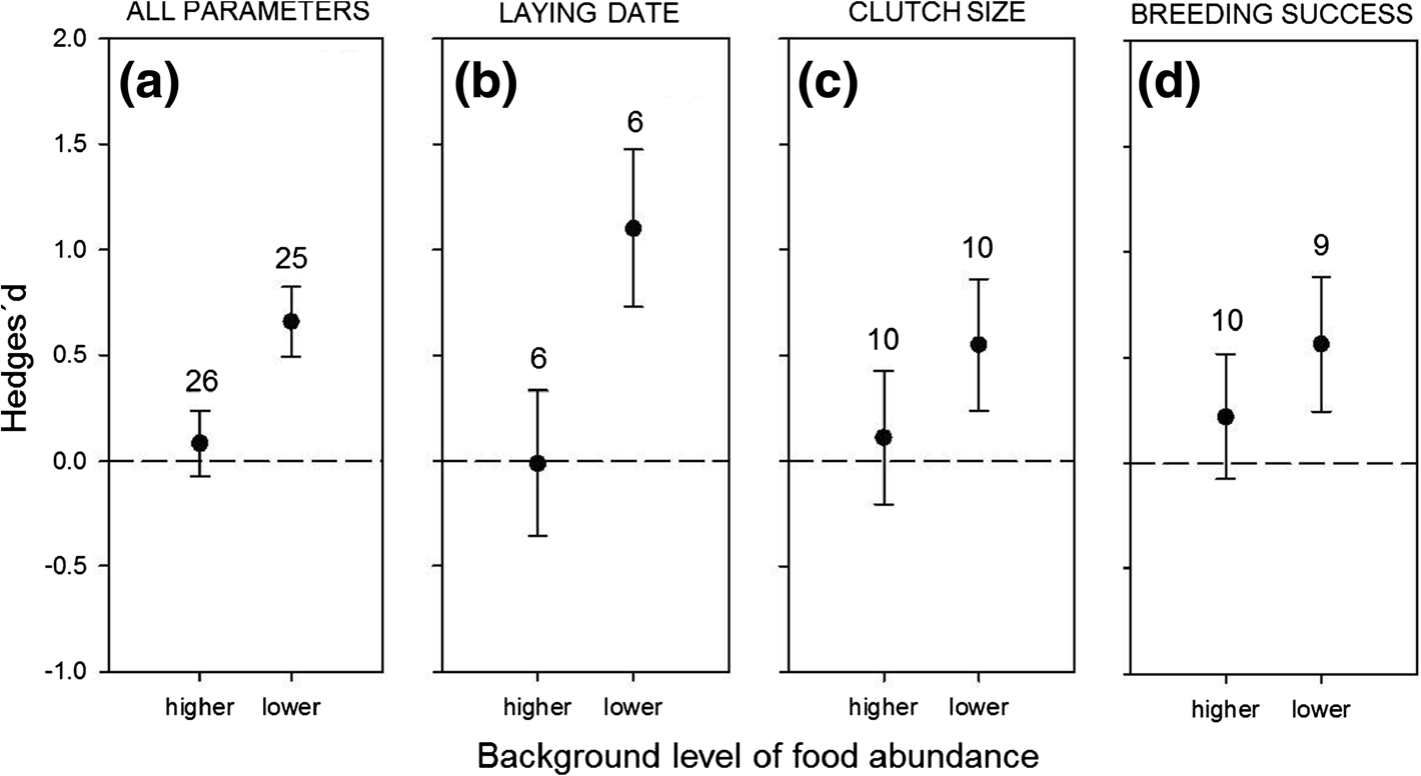
**Effect of the background level of resources on reproductive responses of birds.** Effect of the background level of resources in the environment (higher/lower than average) on variation in **(a)** three bird reproductive parameters, including **(b)** laying date, **(c)** clutch size and **(d)** breeding success in response to food supplementation experiments. Mean effect sizes (Hedges´*d*) and 95% confidence intervals are presented, with sample size (number of experiments) above bars.

After controlling for the type of the reproductive parameter measured, food supplementation produced larger increases in reproductive responses when the background level of resources was lower than average, while supplementation had no detectable effect when the level of resources was higher than average (*Q* = 35.6; *d.f.* = 3; *P* < 0.0001). This effect was detectable in all three reproductive parameters (laying date, clutch size, breeding success; Figure 5).

## Discussion

We aimed to synthesise the outcomes of food supplementation experiments in several bird reproductive parameters by using quantitative meta-analyses. We found mostly positive effects of food supplementation experiments on bird reproduction, indicating that within the pool of bird species and environments reviewed, extra food is often allocated to immediate reproduction. The effects of supplementary food were still variable across reproductive parameters; this is consistent with the idea that varying energetic costs are associated with different stages of the breeding cycle, and it also shows that bird species have evolved different life-history strategies to cope with environmental variability in food supply. The average increase in bird breeding success (response ratio: ${\overline{\mathrm{X}}}_{\left(\mathrm{E}\mathrm{X}\mathrm{P}\right)}/{\overline{\mathrm{X}}}_{\left(\mathrm{CONT}\right)}=1.33$) was of slightly higher magnitude to what Prevedello *et al.* [23] found for the reproductive response of small mammals following food addition (response ratio = 1.19), probably because of the use of different types of reproductive responses (i.e., number of breeding individuals used in Prevedello *et al.* [23] vs. number of offspring in this study). Overall, these positive responses support the idea that both bird and mammal populations can be constrained by food during the energy-demanding period of reproduction. Our analyses revealed that the effects of food supplementation were modulated by (i) environmental factors, such as the background level of food abundance and factors that vary with latitude, (ii) behavioural traits in birds, such as food caching, and (iii) some components of the experimental design, such as the timing of food addition and the level of accessibility to the feed.

Our results showed an overall advancement of laying date in response to food addition across bird taxa, which is in accordance with the results of previous reviews [12,18,20,24]. Breeding earlier generally increases current and future reproductive outputs. For example, it can allow larger clutches [8,25], better chick survival [26,27], more re-nesting attempts [28,29] and more time for the parents to moult and prepare for the winter after breeding [26]. An advancement of egg-laying has also been documented in Europe in relation to increasing average spring temperatures [29-31] that lead to earlier plant growing seasons and forage availability.

We found some support for a latitudinal effect on the advancement of laying dates in response to food supplementation experiments. In accordance with the results of Schoech & Hahn [20], we documented a smaller advancement in egg-laying at higher latitudes. Most birds breeding in northern environments reproduce during a single favourable seasonal window when food is abundant, and they most likely show lower flexibility in life-history stages, such as initiation of egg-laying, than resident birds at lower latitudes. Hence, as suggested by Schoech & Hahn [20], species that rely on endogenous rhythms or environmental stimuli unrelated to food abundance, such as day length at high latitudes, may be limited in their ability to advance their reproduction as an adaptive response to untimely food abundance peaks. However, further research is urgently needed to corroborate these findings since the data currently available are biased against tropical environments. Despite Boutin’s [18] recommendations, to our knowledge only two studies [32,33] have since tested the effect of food addition on the timing of egg-laying in birds below 20°N or 20°S.

Our meta-analyses did not reveal any obvious differences in the effect of food on laying date between migratory and resident species, or between single- and multi-brooded species. There has been an extensive debate over the relative roles of latitude, migratory status and broodedness in modulating the advancement of egg-laying in birds. While the findings of Schoech & Hahn [20] (see also [22]) support the latitudinal gradient hypothesis, Dhondt [21] found no conclusive evidence that latitude influences the response, but identified broodedness as an important factor shaping the response of reproductive timing to food supplementation (see also [24]). These varying results and conclusions among studies probably arise both from the misassignment of some species as multi-brooded in Dhondt [21,22], and the statistical methods used to analyse the data, especially the failure to reduce pseudo-replication and incorporate within-study variances in the calculation of the food supplementation effect. In this paper, we dealt with the statistical issues by (i) controlling for shared phylogeny and study design, (ii) running multi-factor meta-analyses to account for the potential effects of other predictors, (iii) incorporating the within-study variation (SD) into the calculation of the effect sizes, and (iv) weighting the meta-analyses by the inverse of the within-study variance. Although we did not observe any apparent effect of broodedness, the magnitude of food supplementation effects on egg-laying advancement has been shown to differ between single- and multi-brooded species due to their contrasting breeding timing constrains and strategies [24,34]. As only eight experiments on multi-brooded species were available for our meta-analysis, additional experiments are needed to elucidate how broodedness modulates the reproductive response of birds to varying resource abundances in nature.

The effect of food supplementation on the number of eggs laid per clutch showed a moderate positive response. The observed increase in clutch size indicates either that the number of eggs a female can produce in a season is limited by nutrient or energy availability, or that food abundance during egg-laying is a fairly good predictor of resource availability later in the breeding season. In any case, clutch size is believed to be adaptive in many species and should correspond to a trade-off between the physiological constraints of producing more eggs and the energy demands during chick rearing [35]. Indeed, parental survival and future reproductive performance can be reduced due to the increased parental effort needed to rear more chicks to independence [35,36], as has been shown for great tits *Parus major* [37] and jackdaws *Corvus monedula* [38]. Our analyses revealed that food-caching birds, such as magpies, scrub-jays, shrikes, kestrels and some species of tits, increased clutch size in response to food supplementation, while non-caching species did not. Similarly, groups of acorn woodpeckers (*Melanerpes formicivorus*) with acorn stores lay significantly larger clutches than groups that lack stores during the breeding season [39]. Supplemented food can be gathered as soon as it is available and stored for periods when energy demand is higher. Food-caching may enable birds to predict the food conditions later in the season with more accuracy, and as a result stored food may initiate the production of larger clutches. However, it is still unclear whether larger food stores correlate with better overall productivity [40] and the results of our meta-analyses did not allow a conclusion on that matter (see Additional file 1).

The mechanisms driving clutch size variation among individuals, populations and species are complex, as they involve interactions between environmental, behavioural, physiological and genetic determinants. The absence of response of food supplementation in some species (e.g., small passerines, see Additional file 2) may be explained by the inability of the female to use food abundance during egg-laying as a breeding cue. Instead, the female may determine clutch size based on male quality (e.g., [41]), the presence of competitors (e.g., [42]), or by relying on habitat cues that predict environmental conditions and food availability later in the season [43,44]. Moreover, since the number of eggs produced per clutch is often tightly related to the date of egg-laying (i.e., smaller clutches are usually produced later in the season), the response of birds to food supplementation may also differ between early and late breeders in the same population. Unfortunately, we could not test this prediction since average laying dates were in most cases (93%) only provided for the entire breeding season. Finally, there is strong evidence that clutch size has a heritable component in wild populations of several bird species [45] and the use of “animal models” has proven to be valuable to address evolutionary-related questions (e.g., [46,47]).

Studies that delivered supplemental food merely during the pre-laying and/or egg-laying periods showed, on average, no detectable increase in breeding success (i.e., number of fledglings produced), whereas studies where food was added from hatching to fledging or throughout the breeding season documented net increases in breeding success. Supplementing food during pre-laying or egg-laying may actually create a bottle-neck for the birds since the advancement of egg-laying could create a mismatch between the nestling period and the maximal availability of nestling-suitable foods [16], which in turn increases the cost for the parents during chick rearing. However, as shown by von Brömssen & Jansson [26], the supplementation of food during pre-laying can also improve bird fitness in a different way, i.e. food-supplemented parents do not increase the absolute number of offspring produced but are able to produce individual offspring that are in better condition than young of non-supplemented parents.

Boutin [18] argued that food supplementation experiments should account for the possibility that the observed population responses, or the lack thereof, can merely be the result of competition and/or ineffective food delivery. Here, we documented the absence of a detectable effect of food supplementation on the clutch size and breeding success of birds when the target species did not have full access to the food provided, due to interactions at the feeding stations with con- or hetero-specifics. Manipulating food supply can attract a variety of species, both birds and mammals, from outside of the focal experimental area. When food is limiting, competition is likely to increase and can prevent subordinate individuals or species from utilising the feed. Based on our results, the risk of competition for the supplementary food tended to be low in those bird species that show strong territoriality or aggressive behaviour towards intruders, and in cavity-nesting species, to which food can be delivered inside nest boxes. We found that corvids were more likely to increase their breeding success in response to food supplementation than other bird groups (see Additional file 2), probably because corvids often exclude other birds from their territories and thereby from established feeders [48], but also because they can use novel food sources exhaustively due to their strong cognitive abilities and opportunist foraging behaviour.

Food supplementation increased bird reproductive responses when the background level of resources was lower than average, while supplementation had no detectable effect when food was unusually abundant in nature. The threshold hypothesis predicts that only individuals experiencing background levels of food availability below a saturation point will respond to supplementary feeding [18,25]. The relationship between food availability and reproductive traits may thus be non-linear and other factors, such as predation or competition for territories, become limiting when the background level of food is high [1,28,49]. The reproductive decisions of individuals in a given population may vary between years due to changing environmental conditions. This highlights the importance of measuring natural food abundance during any supplementation experiment as well as the value of conducting experiments over multiple years in varying environmental conditions.

### Conclusions and future prospects

Our results confirm that food supply plays a crucial role in avian ecology and evolution, through its effects on individual behaviours and life-history traits. They also imply that the extent of food limitation varies across the multiple stages of bird reproductive cycle, but also across bird taxa and environmental conditions. Latitude appears to modulate the effect of food supplementation on bird reproduction: birds living at low latitudes may be more plastic in advancing their timing of breeding, as compared to birds breeding at higher latitudes where factors other than food may constrain life-history parameters. On the other hand, it has been suggested (in line with the “slow pace of life in the tropics” syndrome [50]) that tropical resident birds should invest extra energy into self-maintenance rather than in immediate reproductive costs [51], and the rare manipulative experiments to address the question of resource allocation trade-offs in the tropics have produced contrasting results (e.g., [51,52]). Certainly, more food supplementation studies in tropical and equatorial environments are needed to be able to conclude on that matter. Comparative studies of closely-related bird species breeding in different environments will also help understanding how resource allocation changes along a latitudinal gradient.

Our findings further revealed that the results of food supplementation experiments could be affected by inadequate experimental designs. This is directly relevant to situations where food supplementation is applied as a conservation tool to boost the breeding performance of endangered birds. For example, if the objective of a supplementary feeding program is to enhance the overall productivity of a bird population, we do not recommend delivering the food during the pre-laying or egg-laying period only as it can create a bottle-neck for the parents later in the breeding season, if the abundance of resources in the environment does not match the energy needs of the parents for rearing more chicks. Instead, we encourage conservation managers to extend the food delivery until the hatching or chick-rearing period. Furthermore, because food supplementation can alter the interactions between individuals and species, it is crucial to ensure that the risk of competition for the supplemented feed is limited. In species where males tend to monopolise the feeders, this issue can be mitigated either by using large numbers of feeders (e.g., ***>***3 per territory) so that females can have unrestricted access to supplemental food, or by delivering the feed to the females directly. Moreover, delivering the food over large treatment areas (with small perimeter-area ratios) instead of small clumped areas, can reduce the flow of intruders and hence the relative effect of immigration (e.g., [23]). Preventing non-target species or conspecifics from monopolising the supplemented food can be achieved by using feeders designed to supplement the feed to experimental animals only (e.g., automated feeders, [53]). Given that the feed is often delivered *ad libitum* over the entire experimental area, it can be difficult to identify which animals are utilising it and, hence, assess the effect of food on life-history parameters, such as reproduction, growth, condition, at the individual level. Recently, Robb *et al*. [54] have pointed out the potential for stable isotopes to trace the use of food supplements in animals and measure the variation in food use between recipient individuals. Alternatively, the use of radio frequency identification systems (RFID) can be useful in quantifying bird visits to feeders [55]. Further research is also needed to test how the spatial dispersion of supplementary food (e.g., patchy vs. scattered; [56]) affects intra- and inter-specific competition and, hence, the outcome of the food supplementation experiment. Supplementing endangered populations should, however, always be applied with caution as some recent studies have warned against potential adverse consequences of food supplementation on nestling health [57] and sex-ratio [58], breeding success [59], and the carry-over effects of winter food provision on the investment in egg production [60].

Another major issue related to the experimental design is the nutritional quality of the feed, which unfortunately could not be addressed in this review due to the lack of data provided by the studies included, but should be cautiously considered in future studies. Growing evidence suggests that the ecological impacts of food supplementation can depend on the specific nutritional profile of provisioned foods [48,60-64] and that the energetic content of the food (in joules or calories) does not necessarily constitute an indicator of food quality [61,63]. For example, the availability of essential amino acids [62,65] and vitamins or antioxidants [60] may constrain egg production in birds. The geometric framework for nutrition (see, e.g., [66]) explores how animals simultaneously regulate the intake of multiple nutrients, and has recently been applied in birds [67]. This modelling approach contributes further understanding of the relationships between the nutritional ratios of natural food and supplementary feeding, and reproduction in birds, and hence can assist with devising effective supplementary food to endangered species [67].

Despite the large body of scientific literature on the impacts of food supply in bird life-history traits and reproductive strategies, some groups of birds and types of environments remain severely under-studied. Large-scale supplementation experiments, testing the effect of food addition on several populations or species of the same groups of birds in variable climates or environmental conditions, would be highly desirable. Finally, despite the accumulation of food supplementation studies over the past thirty years, most of this research has focused on immediate reproductive investments during the current breeding season; very little is known about the carry-over effects of food on offspring and parental survival when supplementation is finished, and about how food limitation may constrain future reproduction (but see [68]).

## Materials and methods

### Literature search

We searched for studies that tested the effects of experimental food supplementation on reproduction of birds by conducting a literature search on the Web of Science and Google Scholar using combinations of the following keywords: “experiment*”, “manipulat*”, “food”, “supplement*”, “feeding”, “addition”, “bird”, “reproduction” and “population”. We also screened the reference lists of all retrieved articles and previous reviews (e.g., [12,18,20]) for other relevant publications. Searching ended in June 2013.

**Figure 6:**
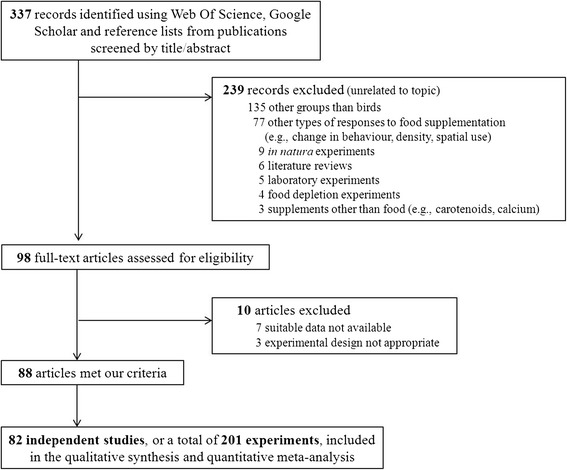
**Flow chart detailing the review and data selection process.** Flow chart detailing the process of record collection and study elimination for the qualitative synthesis and quantitative meta-analyses of food supplementation effects on bird reproduction. Several articles can be considered as a single independent study if they share the same experimental design and were conducted in the same study area.

We restricted our search to food manipulations that provided sufficient data to calculate an effect size. When the means, error measurements or sample sizes were not provided in the publication, the authors were contacted. We discarded studies that reported the reproductive parameters of animals feeding in refuse dumps or of animals studied in habitats of different quality (with no food addition), as well as studies admitting that the supplemented food was not sufficient in either quality or quantity to adequately test for the effects of food supplementation. Out of 337 records identified, a total of 82 independent studies met our criteria (including 201 experiments). Data obtained directly from the authors of the reviewed publications amounted to 8% (16 experiments) of the whole dataset. Three of the authors (LR, EKoivisto, PS) extracted all data from the text, tables and figures (using DigitizeIt software) of the original publications. The process of record collection and study exclusion for the qualitative synthesis and quantitative meta-analyses is detailed in a PRISMA-type flow chart (Figure 6).

### Variables and effect sizes

The experiments were scored for variables that related to the behaviour and life-history traits of each study species by collating data from original publications or other literature (i.e., bird handbooks, internet; see Additional file 3 for more details). Each species was assigned to one of the six established bird types (corvids, passerines other than corvids, birds of prey, seabirds, wetland birds, others; see Additional file 3 for details on the categories). Behavioural variables included migratory status (migratory/year-round resident), food caching (caching/non-caching) and propensity to accept a new type of food (generalist diet/specialist diet), while life-history traits measured maximal clutch size, broodedness (one/more than one clutch per year) and body mass. All experiments were also scored for variables related to the experimental setup, such as timing of food supplementation (before and/or during egg-laying only/other timings, i.e., from hatching to fledging or throughout the breeding season), the level of accessibility to the feed for the target species (low/intermediate/high), environmental conditions, such as latitude and elevation (m a.s.l.) of the study site, and the relative background level of food resources (higher/lower than average) (see Additional file 3). The background level of resources was assigned for all studies that estimated the abundance of the most common food item or prey for the target species or reported clear differences in environmental quality (i.e., due to drastic changes in weather conditions) between study years. Food accessibility was considered low when food was substantially exploited by dominants, immigrants and/or non-target species. Accessibility was deemed high when supplemented food was delivered to the nest or nest box, or to the adults directly, and when direct observations indicated that most or all of the food was consumed by the target species. Accessibility was considered intermediate when food was only occasionally exploited by dominants, immigrants and/or non-target species, and not provided directly to the nest or nest box, or to the adults.

The reproductive responses considered in our analyses included laying date, clutch size, egg size, hatching success, brood size, chick body mass and breeding success. Hatching success was defined as the number of hatchlings divided by the total number of eggs laid. Several estimates of breeding success were used, i.e. the number of fledglings per nest, the number of fledglings divided by the total number of hatchlings or by the number of pairs that initiated reproduction or successfully fledged young, if the number of hatchlings was not reported.

Frequently, ecologists are interested in the actual difference in mean performance or the proportional change in performance in response to a treatment. This is why Hedges’*d* and the log response ratio (ln *R*) have been so commonly used as effect sizes in ecological meta-analyses. Since this study is interested in the factors causing variation in life-history traits of bird species, we need to incorporate a measure of variance in the calculation of the effect size, so that a change in any reproductive parameter can be interpreted as the fitness benefit to that species. For example, clutch size might be very constrained in one species and thus a 10% change in response to food supplementation (e.g., change from 1 ± 0.5 SD to 1.1 ± 0.5 SD) is to be valued more than a 10% change when clutch size is less constrained (e.g., change from 1 ± 1 SD to 1.1 ± 1 SD).

We used the unbiased standardized mean difference Hedges’*d* (hereafter *d*) as a measure of effect size in our meta-analyses. This effect size can be interpreted as the difference between the reproductive responses of supplemented birds versus non-supplemented birds, measured in units of standard deviations. Thus, large differences and low variability generate the largest effect sizes [69,70]. Positive *d* values indicate that food supplementation has a positive effect on the reproduction of a study species, while negative values indicate greater values in controls compared to the experimental group. By convention, a large effect is indicated by |*d|* > 0.8, a moderate effect by |*d|* = 0.2-0.8, and a small effect by |*d|* = 0-0.2 [71,72].

### Analyses

We used the metafor package [73] in R 3.0.2 to perform the meta-analyses and estimate a pooled effect size for each reproductive parameter and explanatory variable. In all meta-analyses, we controlled for repeated observations on the same species from the same study area by including Study ID as a random variable in the models. We also controlled for correlated structures due to shared phylogenetic history by specifying a phylogenetic correlation matrix of the species included in the meta-analysis and including Species as a random variable in our models (see Additional file 4 for the phylogenetic bird trees used and their characteristics). Variation in response magnitudes among bird types was confirmed by the heterogeneity statistics *Q* computed on the laying date, clutch size and breeding success datasets (Additional file 2). The following additional procedures also helped minimising the risk of pseudo-replication in the datasets: (i) each type of reproductive parameter was analysed separately, thereby avoiding the inclusion of several parameters taken from the same species or study area in the same dataset, (ii) in cases where the same reproductive parameters were measured on the same species over several years, an average effect size was calculated over all study years, (iii) in cases where a study tested different food qualities or supplementation timings on the same species, we selected the experiment with the highest effect size. In all meta-analyses, effect sizes were weighted by the inverse of their within-variance. Mean effect sizes showed significant effects of food addition when their 95% confidence intervals did not overlap zero. Model convergence was checked by inspecting the profile of the maximum restricted log-likelihood of the full models.

First, we estimated the overall effects of food supplementation on seven reproductive parameters, namely laying date (n = 41 experiments), clutch size (n = 46), egg size (n = 26), hatching success (n = 18), brood size (n = 5), chick body mass (n = 21) and breeding success (n = 38), by performing weighted random-effect meta-analyses without fixed explanatory variables (i.e., null random models). Second, we tested the effect of several explanatory variables on laying date, clutch size and breeding success (i.e., parameters for which *N* ≥ 30), separately. For laying date, we tested the effect of migratory status, broodedness, diet, accessibility to the feed, latitude and elevation. Meta-analyses for clutch size tested the effect of caching behaviour, maximal clutch size, body mass, accessibility to the feed, diet and latitude. Explanatory variables for breeding success included caching behaviour, accessibility to the feed, timing of food addition, diet and body mass. For each analysis, we constructed a set of models that included all possible combinations of explanatory variables. We used conditional Akaike’s Information Criterion, corrected for small sample size (AICc), to compare the goodness of fit among models. Models were ranked in relation to each other using ∆AICc values. AICc weights (*w*_*i*_) were calculated to assess the relative likelihood of each model considered. Finally, we used multi-model inference to quantify the relative explanatory importance of variables over the set of candidate models. Variable weights were calculated as the sum of AICc weights of all the models including a given variable.

The background level of resources can affect the magnitude of the bird response to food supplementation (e.g., [25]; see also Results) but only 18 studies (out of 82) monitored or commented on the natural resource levels in their study area for the whole study period. For the 41 studies conducted over several years, we remained conservative in our approach and calculated an average effect size over the whole study period. Studies that were performed within one year and did not report the background level of resources (*n* = 38) were assumed to be conducted under average environmental conditions. Three studies reported experiments only at a persistently and distinctly lower [74] (poor quality habitat with low prey densities) or higher [75,76] (peak of main prey abundance in rodent specialised predators) background level of resources than average and were discarded to avoid biasing the analyses. Therefore, we made sure that the effect sizes used in all the above-mentioned meta-analyses matched averaged environmental conditions. The final sample size for the analyses described above was 195 experiments from 79 studies (Additional file 3).

Third, we explored the effect of varying background levels of food resources (lower/higher than average, as stated in the original publications) on the success of food supplementation in a separate meta-analysis. Reproductive parameter (laying date, clutch size, breeding success) was included as a fixed covariate in the meta-analysis since responses to food supplementation varied across reproductive parameters (see Results). This dataset consisted of 51 experiments from 18 studies (Additional file 3).

Finally, we examined the possibility of publication bias in our datasets by generating funnel and normal quantile plots (Additional file 5).

### Availability of supporting data

The data sets supporting the results of this article are included within the article (and its additional files).

## Additional files

Additional file 1:
**Model selection procedure and mean effect sizes of the variables that did not show any support in explaining variation in the datasets.**
Additional file 2:
**Results of the meta-analyses exploring the effect of food supplementation on laying date, clutch size and breeding success across several bird types.**
Additional file 3:
**List of all the publications included in the meta-analyses testing the effect of food supplementation on several reproductive parameters.**
Additional file 4:
**Phylogenetic trees used in the phylogenetic meta-analyses.**
Additional file 5:
**Examination of publication bias.**
