# Supplementary material for: Reproductive responses of birds to experimental food supplementation: a meta-analysis
Source: Front Zool. 2014 Oct 31;11:80. doi: 10.1186/s12983-014-0080-y (PMC4222371; doi:10.1186/s12983-014-0080-y)
Supplement: Additional file 1: — Model selection procedure and mean effect sizes of the variables that did not show any support in explaining variation in the datasets. [file 12983_2014_80_MOESM1_ESM.docx]

**Additional file 1. Model selection procedure and mean effect sizes of the variables that did not show any support in explaining variation in the datasets**

**1.A.a*.*** *Performance of models explaining variation in laying date in response to food supplementation*

|  | **Model** | **log likelihood** | **AICc** | **ΔAICc** | ***wi*** |
| --- | --- | --- | --- | --- | --- |
| 1 | rma.mv(d~Lat+FoodAccess) | -37.02 | 88.84 | 0.00 | 0.13 |
| 2 | rma.mv(d~Lat) | -40.44 | 90.06 | 1.22 | 0.07 |
| 3 | rma.mv(d~Lat+Elevt+FoodAccess) | -36.10 | 90.20 | 1.36 | 0.07 |
| 4 | rma.mv(d~Lat+FoodAccess+Migra+Brood) | -36.13 | 90.26 | 1.42 | 0.07 |
| 5 | rma.mv(d~Lat+FoodAccess+Migra) | -36.13 | 90.26 | 1.42 | 0.07 |
| 6 | rma.mv(d~Lat+Elev) | -39.35 | 90.58 | 1.74 | 0.06 |
| 7 | rma.mv(d~Lat+FoodAccess+Diet) | -36.48 | 90.97 | 2.13 | 0.05 |
| 8 | rma.mv(d~Lat+Migra) | -39.64 | 91.16 | 2.32 | 0.04 |
| 9 | rma.mv(d~Lat+Diet) | -39.73 | 91.34 | 2.50 | 0.04 |
| 10 | rma.mv(d~Lat+FoodAccess+Brood) | -36.70 | 91.39 | 2.55 | 0.04 |
| 11 | rma.mv(d~Lat+Brood) | -39.86 | 91.60 | 2.76 | 0.03 |
| 12 | rma.mv(d~Lat+Elev+Migra) | -38.65 | 92.10 | 3.26 | 0.03 |
| 13 | rma.mv(d~Lat+Diet+Elev) | -38.66 | 92.12 | 3.28 | 0.03 |
| 14 | rma.mv(d~Lat+FoodAccess+Migra+Elev) | -35.35 | 92.25 | 3.41 | 0.02 |
| 15 | rma.mv(d~Lat+Brood+Elev) | -38.73 | 92.27 | 3.43 | 0.02 |
| 16 | rma.mv(d~Lat+Diet+FoodAccess+Elev) | -35.54 | 92.61 | 3.77 | 0.02 |
| 17 | rma.mv(d~Lat+Diet+Migra) | -38.91 | 92.62 | 3.78 | 0.02 |
| 18 | rma.mv(d~Lat+FoodAccess+Migra+Diet) | -35.57 | 92.68 | 3.84 | 0.02 |
| 19 | rma.mv(d~Lat+Migra+Brood) | -39.10 | 93.00 | 4.16 | 0.02 |
| 20 | rma.mv(d~Lat+FoodAccess+Brood+Elev) | -35.77 | 93.08 | 4.24 | 0.02 |
| 21 | rma.mv(d~Lat+Diet+Brood) | -39.23 | 93.26 | 4.42 | 0.01 |
| 22 | rma.mv(d~Lat+Diet+Brood+FoodAccess) | -36.13 | 93.79 | 4.95 | 0.01 |
| 23 | rma.mv(d~Lat+Diet+Migra+Elev) | -37.93 | 93.87 | 5.03 | 0.01 |
| 24 | rma.mv(d~Lat+Brood+Elev+Migra) | -38.08 | 94.16 | 5.32 | 0.01 |
| 25 | rma.mv(d~Lat+Diet+Brood+Elev) | -38.11 | 94.23 | 5.39 | 0.01 |
| 26 | rma.mv(d~Migra) | -42.71 | 94.59 | 5.75 | 0.01 |
| 27 | rma.mv(d~Lat+Diet+Brood+Migra) | -38.43 | 94.87 | 6.03 | 0.01 |
| 28 | rma.mv(d~Lat+FoodAccess+Migra+Elev+Diet) | -34.77 | 95.04 | 6.20 | 0.01 |
| 29 | rma.mv(d~Diet+Migra) | -41.75 | 95.38 | 6.54 | 0.01 |
| 30 | rma.mv(d~1) | -44.36 | 95.38 | 6.54 | 0.01 |
| 31 | rma.mv(d~Lat+FoodAccess+Migra+Brood+Elev) | -35.00 | 95.51 | 6.67 | 0.00 |
| 32 | rma.mv(d~Diet) | -43.23 | 95.64 | 6.80 | 0.00 |
| 33 | rma.mv(d~Brood+Migra) | -41.93 | 95.74 | 6.90 | 0.00 |
| 34 | rma.mv(d~Brood) | -43.33 | 95.83 | 6.99 | 0.00 |
| 35 | rma.mv(d~Lat+Diet+Brood+Elev+FoodAccess) | -35.18 | 95.86 | 7.02 | 0.00 |
| 36 | rma.mv(d~Lat+FoodAccess+Migra+Diet+Brood) | -35.19 | 95.88 | 7.04 | 0.00 |
| 37 | rma.mv(d~FoodAccess+Migra) | -40.60 | 96.00 | 7.16 | 0.00 |
| 38 | rma.mv(d~Elev+Migra) | -42.13 | 96.13 | 7.29 | 0.00 |
| 39 | rma.mv(d~FoodAccess) | -42.14 | 96.15 | 7.31 | 0.00 |
| 40 | rma.mv(d~Lat+Diet+Brood+Elev+Migra) | -37.42 | 96.39 | 7.55 | 0.00 |
| 41 | rma.mv(d~Diet+FoodAccess) | -40.90 | 96.60 | 7.76 | 0.00 |
| 42 | rma.mv(d~Elev) | -43.73 | 96.65 | 7.81 | 0.00 |
| 43 | rma.mv(d~Diet+Brood) | -42.42 | 96.72 | 7.88 | 0.00 |
| 44 | rma.mv(d~FoodAccess+Migra+Diet) | -39.47 | 96.94 | 8.10 | 0.00 |
| 45 | rma.mv(d~Diet+Brood+Migra) | -41.08 | 96.97 | 8.13 | 0.00 |
| 46 | rma.mv(d~Diet+Elev) | -42.60 | 97.07 | 8.23 | 0.00 |
| 47 | rma.mv(d~Diet+Migra+Elev) | -41.15 | 97.11 | 8.27 | 0.00 |
| 48 | rma.mv(d~Brood+Elev) | -42.77 | 97.42 | 8.58 | 0.00 |
| 49 | rma.mv(d~Elev+Brood+Migra) | -41.38 | 97.57 | 8.73 | 0.00 |
| 50 | rma.mv(d~Brood+FoodAccess) | -41.47 | 97.73 | 8.89 | 0.00 |
| 51 | rma.mv(d~Elev+FoodAccess) | -41.48 | 97.77 | 8.93 | 0.00 |
| 52 | rma.mv(d~FoodAccess+Migra+Elev) | -39.96 | 97.92 | 9.08 | 0.00 |
| 53 | rma.mv(d~FoodAccess+Migra+Brood) | -40.05 | 98.09 | 9.25 | 0.00 |
| 54 | rma.mv(d~Diet+Brood+Elev) | -41.85 | 98.50 | 9.66 | 0.00 |
| 55 | rma.mv(d~Diet+FoodAccess+Elev) | -40.26 | 98.52 | 9.68 | 0.00 |
| 56 | rma.mv(d~FoodAccess+Migra+Elev+Brood+Diet+Lat) | -34.39 | 98.77 | 9.93 | 0.00 |
| 57 | rma.mv(d~Diet+Brood+FoodAccess) | -40.39 | 98.78 | 9.94 | 0.00 |
| 58 | rma.mv(d~Diet+Brood+Elev+Migra) | -40.52 | 99.05 | 10.21 | 0.00 |
| 59 | rma.mv(d~FoodAccess+Migra+Elev+Diet) | -38.81 | 99.16 | 10.32 | 0.00 |
| 60 | rma.mv(d~FoodAccess+Migra+Diet+Brood) | -38.99 | 99.53 | 10.69 | 0.00 |
| 61 | rma.mv(d~Elev+Brood+FoodAccess) | -40.86 | 99.73 | 10.89 | 0.00 |
| 62 | rma.mv(d~FoodAccess+Migra+Elev+Brood) | -39.44 | 100.42 | 11.58 | 0.00 |
| 63 | rma.mv(d~Diet+Brood+Elev+FoodAccess) | -39.78 | 101.09 | 12.25 | 0.00 |
| 64 | rma.mv(d~FoodAccess+Migra+Elev+Brood+Diet) | -38.36 | 102.21 | 13.37 | 0.00 |


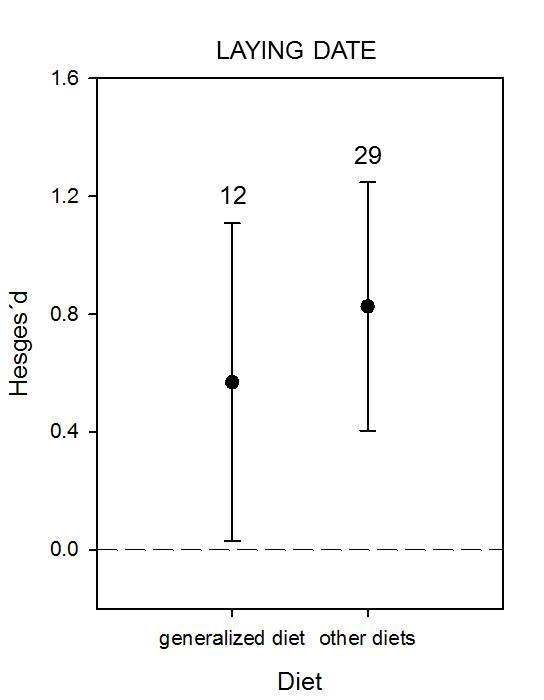

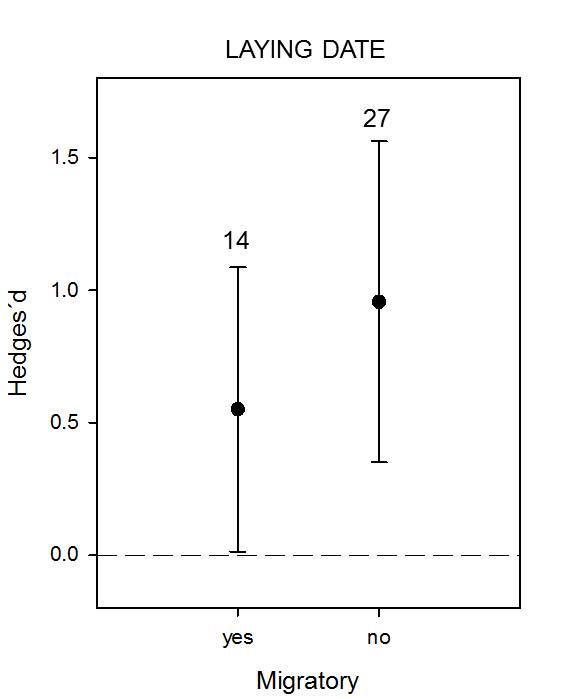

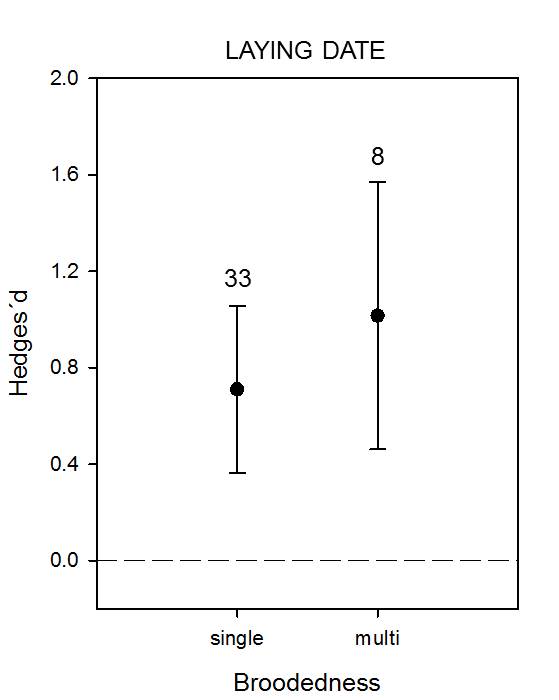
**1.A.b.** *Mean effect sizes of the variables that did not explain variation in laying date*


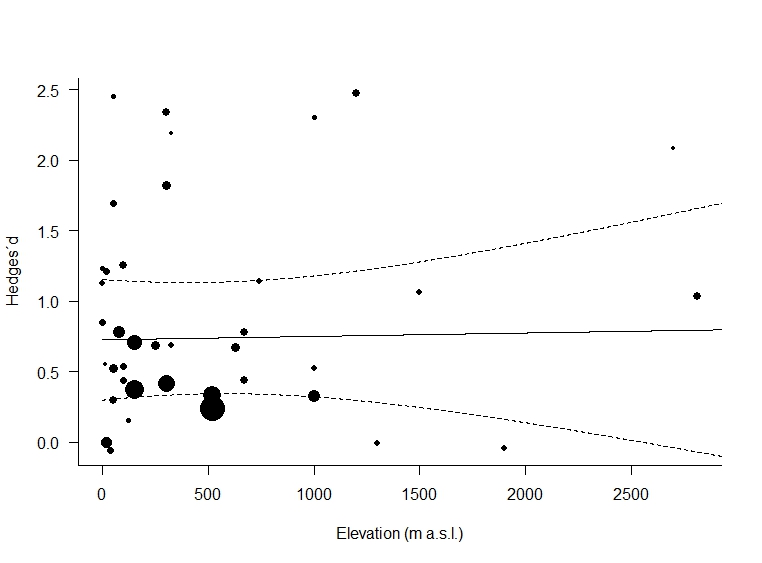

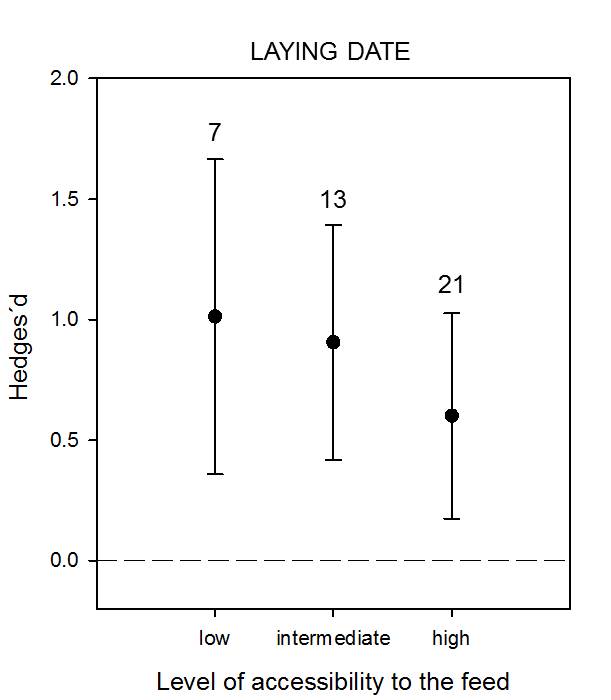


**1.B.a.** *Performance of models explaining variation in clutch size in response to food supplementation*

|  | **Model** | **log likelihood** | **AICc** | **ΔAICc** | ***wi*** |
| --- | --- | --- | --- | --- | --- |
| 1 | rma.mv(d~FoodAccess+FoodCach) | -26.77 | 67.94 | 0.00 | 0.13 |
| 2 | rma.mv(d~FoodAccess+FoodCach+MaxClutch) | -25.72 | 68.84 | 0.90 | 0.08 |
| 3 | rma.mv(d~FoodCach+MaxClutch) | -28.68 | 68.98 | 1.04 | 0.08 |
| 4 | rma.mv(d~FoodAccess) | -28.75 | 69.13 | 1.19 | 0.07 |
| 5 | rma.mv(d~FoodAccess+FoodCach+Lat) | -26.32 | 70.04 | 2.10 | 0.04 |
| 6 | rma.mv(d~FoodAccess+Lat) | -27.97 | 70.35 | 2.41 | 0.04 |
| 7 | rma.mv(d~FoodAccess+FoodCach+MaxClutch+Lat) | -24.86 | 70.37 | 2.43 | 0.04 |
| 8 | rma.mv(d~FoodAccess+FoodCach+Mass) | -26.58 | 70.56 | 2.62 | 0.03 |
| 9 | rma.mv(d~FoodCach) | -30.78 | 70.58 | 2.64 | 0.03 |
| 10 | rma.mv(d~FoodAccess+MaxClutch) | -28.10 | 70.61 | 2.67 | 0.03 |
| 11 | rma.mv(d~FoodAccess+FoodCach+Diet) | -26.64 | 70.67 | 2.73 | 0.03 |
| 12 | rma.mv(d~FoodAccess+Mass) | -28.21 | 70.82 | 2.88 | 0.03 |
| 13 | rma.mv(d~FoodCach+MaxClutch+Lat) | -28.34 | 71.09 | 3.15 | 0.03 |
| 14 | rma.mv(d~FoodAccess+MaxClutch+Lat) | -26.87 | 71.13 | 3.20 | 0.03 |
| 15 | rma.mv(d~FoodHoard+MaxClutch+Mass) | -28.52 | 71.44 | 3.50 | 0.02 |
| 16 | rma.mv(d~FoodCach+MaxClutch+Diet) | -28.52 | 71.44 | 3.50 | 0.02 |
| 17 | rma.mv(d~FoodAccess+FoodCach+MaxClutch+Mass) | -25.46 | 71.56 | 3.62 | 0.02 |
| 18 | rma.mv(d~FoodAccess+Diet) | -28.61 | 71.62 | 3.68 | 0.02 |
| 19 | rma.mv(d~FoodAccess+FoodCach+MaxClutch+Diet) | -25.60 | 71.85 | 3.91 | 0.02 |
| 20 | rma.mv(d~FoodAccess+MaxClutch+Mass) | -27.26 | 71.91 | 3.97 | 0.02 |
| 21 | rma.mv(d~FoodHoard+Mass) | -30.35 | 72.32 | 4.38 | 0.01 |
| 22 | rma.mv(d~FoodCach+Diet) | -30.55 | 72.72 | 4.78 | 0.01 |
| 23 | rma.mv(d~FoodAccess+Lat+Mass) | -27.67 | 72.73 | 4.79 | 0.01 |
| 24 | rma.mv(d~FoodCach+Lat) | -30.63 | 72.88 | 4.95 | 0.01 |
| 25 | rma.mv(d~FoodAccess+FoodCach+Lat+Mass) | -26.13 | 72.90 | 4.96 | 0.01 |
| 26 | rma.mv(d~FoodAccess+FoodCach+Lat+Diet) | -26.14 | 72.92 | 4.98 | 0.01 |
| 27 | rma.mv(d~FoodAccess+Diet+Lat) | -27.77 | 72.93 | 4.99 | 0.01 |
| 28 | rma.mv(d~FoodAccess+MaxClutch+Diet) | -27.96 | 73.31 | 5.37 | 0.01 |
| 29 | rma.mv(d~FoodAccess+Diet+Mass) | -28.04 | 73.48 | 5.54 | 0.01 |
| 30 | rma.mv(d~FoodAccess+FoodCach+MaxClutch+Lat+Diet) | -24.65 | 73.50 | 5.56 | 0.01 |
| 31 | rma.mv(d~FoodAccess+FoodCach+Diet+Mass) | -26.43 | 73.51 | 5.57 | 0.01 |
| 32 | rma.mv(d~FoodAccess+Mass+MaxClutch+Lat) | -26.44 | 73.53 | 5.59 | 0.01 |
| 33 | rma.mv(d~FoodHoard+Mass+MaxClutch+Lat) | -28.08 | 73.55 | 5.61 | 0.01 |
| 34 | rma.mv(d~FoodAccess+FoodCach+Lat+Mass+MaxClutch) | -24.71 | 73.63 | 5.69 | 0.01 |
| 35 | rma.mv(d~FoodCach+Lat+MaxClutch+Diet) | -28.12 | 73.64 | 5.70 | 0.01 |
| 36 | rma.mv(d~FoodAccess+Lat+MaxClutch+Diet) | -26.64 | 73.92 | 5.98 | 0.01 |
| 37 | rma.mv(d~FoodCach+Diet+Mass+MaxClutch) | -28.33 | 74.04 | 6.11 | 0.01 |
| 38 | rma.mv(d~FoodCach+Diet+Mass) | -30.04 | 74.49 | 6.55 | 0.00 |
| 39 | rma.mv(d~FoodCach+Lat+Mass) | -30.12 | 74.65 | 6.71 | 0.00 |
| 40 | rma.mv(d~FoodAccess+Diet+Mass+MaxClutch) | -27.09 | 74.83 | 6.89 | 0.00 |
| 41 | rma.mv(d~FoodAccess+FoodCach+MaxClutch+Mass+Diet) | -25.33 | 74.87 | 6.93 | 0.00 |
| 42 | rma.mv(d~FoodCach+Diet+Lat) | -30.37 | 75.15 | 7.21 | 0.00 |
| 43 | rma.mv(d~FoodAccess+Diet+Mass+Lat) | -27.46 | 75.57 | 7.63 | 0.00 |
| 44 | rma.mv(d~FoodAccess+FoodCach+Lat+Diet+Mass) | -25.87 | 75.96 | 8.02 | 0.00 |
| 45 | rma.mv(d~FoodCach+Diet+Mass+Lat+MaxClutch) | -27.73 | 76.11 | 8.17 | 0.00 |
| 46 | rma.mv(d~FoodAccess+Diet+Mass+Lat+MaxClutch) | -26.25 | 76.70 | 8.77 | 0.00 |
| 47 | rma.mv(d~FoodCach+Diet+Mass+Lat) | -29.71 | 76.81 | 8.87 | 0.00 |
| 48 | rma.mv(d~MaxClutch) | -33.93 | 76.89 | 8.95 | 0.00 |
| 49 | rma.mv(d~FoodAccess+FoodCach+MaxClutch+Mass+Diet+Lat) | -24.46 | 77.07 | 9.14 | 0.00 |
| 50 | rma.mv(d~1) | -35.58 | 77.74 | 9.80 | 0.00 |
| 51 | rma.mv(d~Mass+MaxClutch) | -33.07 | 77.77 | 9.83 | 0.00 |
| 52 | rma.mv(d~MaxClutch+Lat) | -33.11 | 77.85 | 9.91 | 0.00 |
| 53 | rma.mv(d~Diet+MaxClutch) | -33.55 | 78.73 | 10.79 | 0.00 |
| 54 | rma.mv(d~Mass) | -34.97 | 78.97 | 11.03 | 0.00 |
| 55 | rma.mv(d~Diet) | -35.13 | 79.28 | 11.34 | 0.00 |
| 56 | rma.mv(d~Mass+MaxClutch+Lat) | -32.45 | 79.29 | 11.36 | 0.00 |
| 57 | rma.mv(d~Lat) | -35.21 | 79.45 | 11.51 | 0.00 |
| 58 | rma.mv(d~Diet+Mass+MaxClutch) | -32.73 | 79.85 | 11.91 | 0.00 |
| 59 | rma.mv(d~Diet+Mass) | -34.52 | 80.67 | 12.73 | 0.00 |
| 60 | rma.mv(d~Lat+Mass) | -34.62 | 80.86 | 12.92 | 0.00 |
| 61 | rma.mv(d~Diet+Lat) | -34.64 | 80.90 | 12.96 | 0.00 |
| 62 | rma.mv(d~Diet+Mass+Lat+MaxClutch) | -31.92 | 81.23 | 13.29 | 0.00 |
| 63 | rma.mv(d~Diet+Lat+MaxClutch) | -34.04 | 82.48 | 14.54 | 0.00 |
| 64 | rma.mv(d~Diet+Mass+Lat) | -34.04 | 82.48 | 14.54 | 0.00 |

**1.B.b**. *Mean effect sizes of the variables that did not explain variation in clutch size*


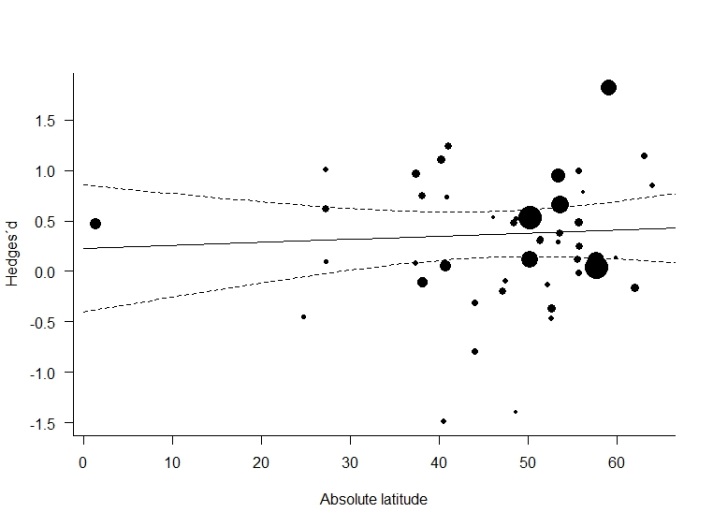
*
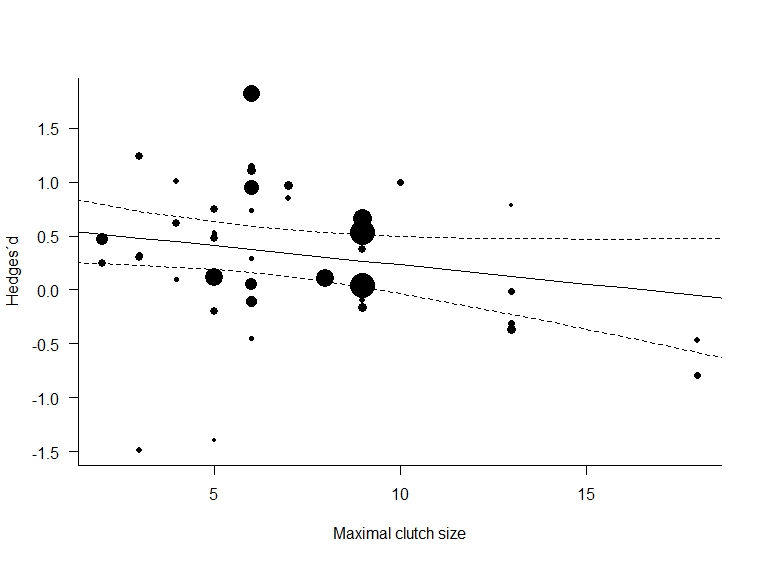

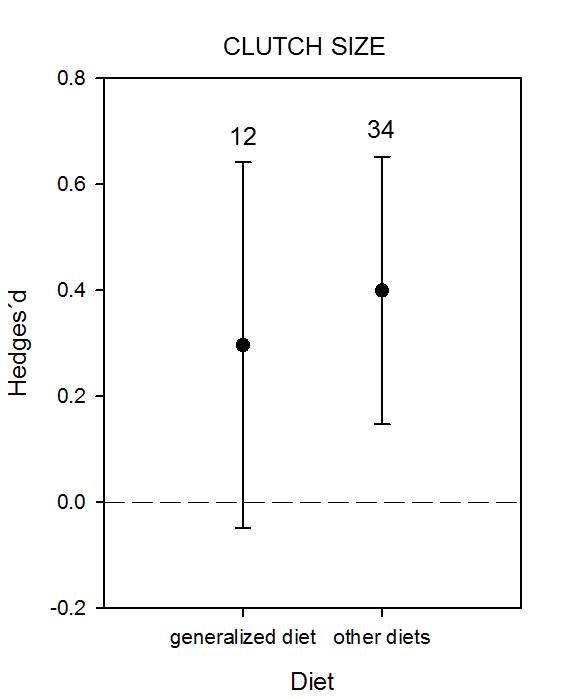
*


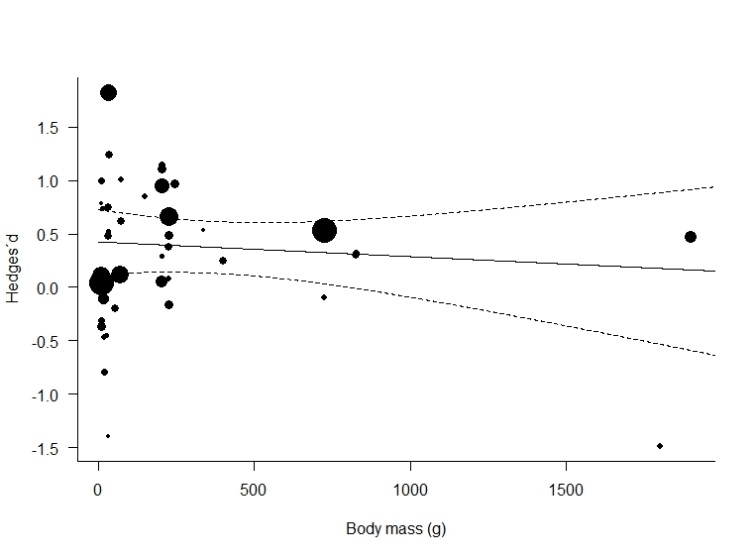


**1.C.a.** *Performance of models explaining variation in breeding success in response to food supplementation*

|  | **Model** | **log likelihood** | **AICc** | **ΔAICc** | ***wi*** |
| --- | --- | --- | --- | --- | --- |
| 1 | rma.mv(d~Timing+FoodAccess) | -39.21 | 93.53 | 0.00 | 0.13 |
| 2 | rma.mv(d~Timing) | -42.14 | 93.57 | 0.04 | 0.12 |
| 3 | rma.mv(d~Timing+Diet) | -40.81 | 93.69 | 0.17 | 0.12 |
| 4 | rma.mv(d~Timing+FoodAccess+Diet) | -37.86 | 94.19 | 0.66 | 0.09 |
| 5 | rma.mv(d~Timing+FoodCach) | -41.40 | 94.86 | 1.33 | 0.06 |
| 6 | rma.mv(d~Timing+Mass) | -41.49 | 95.04 | 1.52 | 0.06 |
| 7 | rma.mv(d~Timing+FoodAccess+FoodCach) | -38.30 | 95.09 | 1.56 | 0.06 |
| 8 | rma.mv(d~Timing+Diet+FoodCach) | -40.04 | 95.19 | 1.67 | 0.05 |
| 9 | rma.mv(d~Timing+Diet+Mass) | -40.18 | 95.47 | 1.94 | 0.05 |
| 10 | rma.mv(d~Timing+FoodAccess+Mass) | -38.60 | 95.68 | 2.15 | 0.04 |
| 11 | rma.mv(d~Timing+FoodAccess+Diet+FoodCach) | -36.79 | 95.85 | 2.32 | 0.04 |
| 12 | rma.mv(d~Timing+FoodCach+Mass) | -40.67 | 96.46 | 2.93 | 0.03 |
| 13 | rma.mv(d~Timing+FoodAccess+Diet+Mass) | -37.12 | 96.50 | 2.97 | 0.03 |
| 14 | rma.mv(d~Timing+Diet+FoodCach+Mass) | -39.37 | 97.23 | 3.70 | 0.02 |
| 15 | rma.mv(d~Timing+FoodAccess+FoodCach+Mass) | -37.64 | 97.53 | 4.01 | 0.02 |
| 16 | rma.mv(d~FoodAccess+Diet) | -41.46 | 98.03 | 4.50 | 0.01 |
| 17 | rma.mv(d~FoodAccess) | -43.02 | 98.11 | 4.58 | 0.01 |
| 18 | rma.mv(d~Timing+FoodAccess+Diet+FoodCach+Mass) | -36.07 | 98.71 | 5.18 | 0.01 |
| 19 | rma.mv(d~FoodAccess+Diet+FoodCach) | -40.21 | 98.89 | 5.36 | 0.01 |
| 20 | rma.mv(d~FoodAccess+FoodCach) | -41.96 | 99.04 | 5.51 | 0.01 |
| 21 | rma.mv(d~FoodAccess+Mass) | -42.33 | 99.76 | 6.23 | 0.01 |
| 22 | rma.mv(d~FoodAccess+Diet+Mass) | -40.71 | 99.89 | 6.36 | 0.01 |
| 23 | rma.mv(d~Diet) | -45.62 | 100.53 | 7.00 | 0.00 |
| 24 | rma.mv(d~FoodAccess+Mass+FoodCach) | -41.22 | 100.92 | 7.39 | 0.00 |
| 25 | rma.mv(d~1) | -47.21 | 101.14 | 7.61 | 0.00 |
| 26 | rma.mv(d~FoodAccess+Diet+FoodCach+Mass) | -39.46 | 101.19 | 7.66 | 0.00 |
| 27 | rma.mv(d~FoodCach+Diet) | -44.74 | 101.55 | 8.02 | 0.00 |
| 28 | rma.mv(d~Diet+Mass) | -44.90 | 101.86 | 8.33 | 0.00 |
| 29 | rma.mv(d~FoodCach) | -46.36 | 102.00 | 8.47 | 0.00 |
| 30 | rma.mv(d~Mass) | -46.38 | 102.04 | 8.51 | 0.00 |
| 31 | rma.mv(d~FoodCach+Mass) | -45.50 | 103.07 | 9.54 | 0.00 |
| 32 | rma.mv(d~Diet+FoodCach+Mass) | -43.99 | 103.10 | 9.57 | 0.00 |

**1.C.b.** *Mean effect sizes of the variables that did not explain variation in breeding success*

*
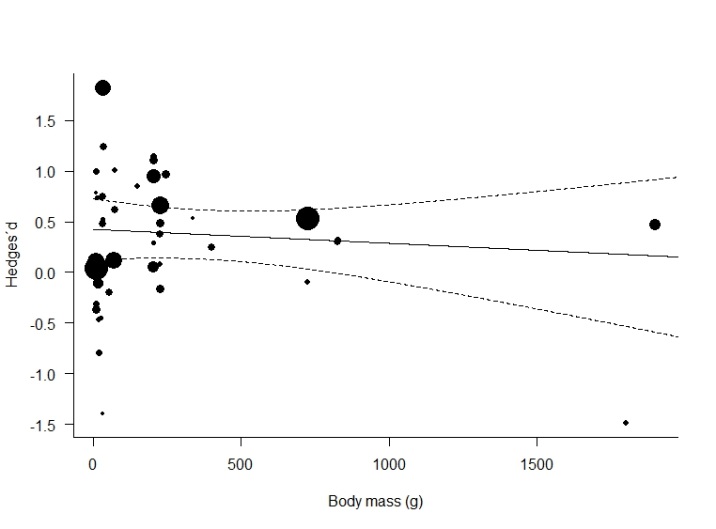
*
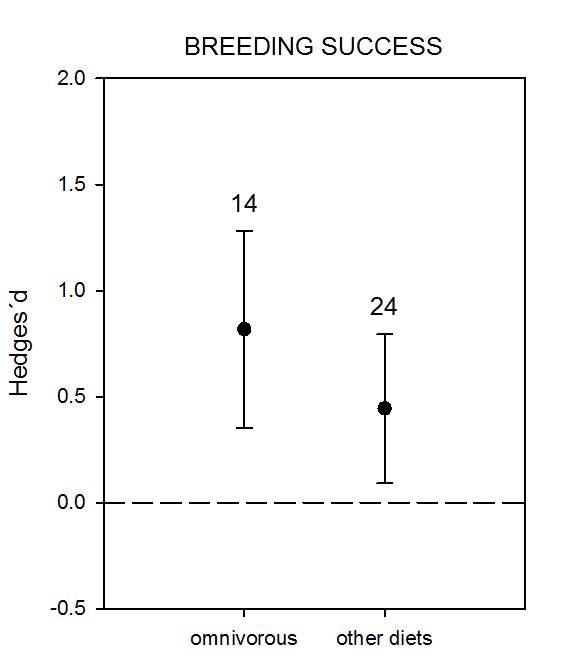
*
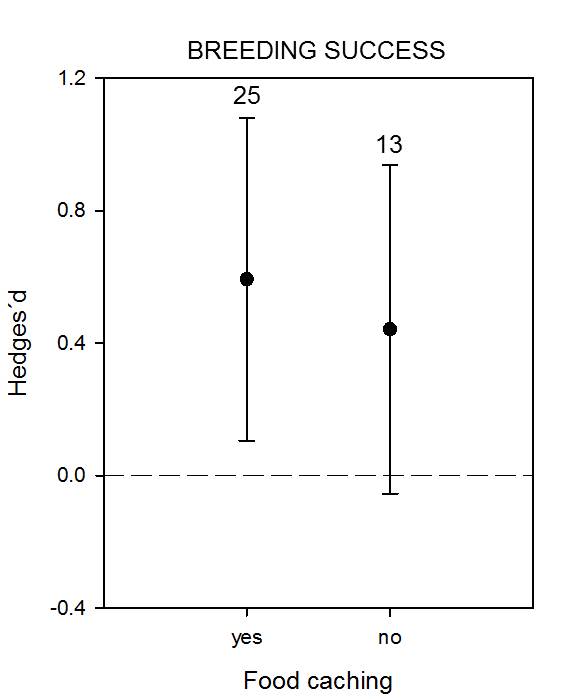
*
