# Supplementary material for: Reproductive responses of birds to experimental food supplementation: a meta-analysis
Source: Front Zool. 2014 Oct 31;11:80. doi: 10.1186/s12983-014-0080-y (PMC4222371; doi:10.1186/s12983-014-0080-y)
Supplement: Additional file 2: — Results of the meta-analyses exploring the effect of food supplementation on laying date, clutch size and breeding success across several bird types. [file 12983_2014_80_MOESM2_ESM.docx]

**Additional file 2**. **Results of the meta-analyses exploring the effect of food supplementation on laying date, clutch size and breeding success across several bird types.**

Heterogeneity statistics *Q*_T_ were first computed in order to estimate the total amount of heterogeneity in each dataset [1]. These statistical tests are analogous to ANOVA, but do not rely on the assumption of homogeneity of variance. When categorical variables, such as bird type, are included in the model, the total heterogeneity *Q*_T_ can be partitioned into between- *Q*_B_ (variance due to the explanatory variables) and within-group *Q*_W_ (variance due to sampling error) components. Model parameters were estimated by the restricted maximum-likelihood method [2]. Mean effect sizes showed significant effects of food addition when their 95% confidence intervals did not overlap zero [1].

Note that the bird types that had sample sizes < 3 studies were excluded from the analyses. This was the case of one wetland bird and three seabirds in the breeding success model, two wetland birds in the clutch size model, and one wetland bird, two seabirds and one other type in the laying date model.

| Variable |  | *n* | Hedges’ *d* | 95% CI | *Q*_B_ | *P*-value | *d.f*. | *Q*_W_ |
| --- | --- | --- | --- | --- | --- | --- | --- | --- |
| LAYING DATE |  |  |  |  |  |  |  |  |
| Bird type |  |  |  |  | 2.9 | 0.24 | 2 | 216.4 |
|  | Small passerines | 19 | 1.248 | 0.833 to 1.663 |  |  |  |  |
|  | Corvids | 9 | 1.025 | 0.286 to 1.763 |  |  |  |  |
|  | Birds of prey | 9 | 0.600 | -0.026 to 1.227 |  |  |  |  |
|  |  |  |  |  |  |  |  |  |
| CLUTCH SIZE |  |  |  |  |  |  |  |  |
| Bird type |  |  |  |  | 4.4 | 0.23 | 3 | 163.8 |
|  | Birds of prey | 8 | 0.587 | 0.207 to 0.967 |  |  |  |  |
|  | Corvids | 9 | 0.470 | 0.136 to 0.804 |  |  |  |  |
|  | Seabirds | 4 | 0.331 | -0.118 to 0.779 |  |  |  |  |
|  | Small passerines | 23 | 0.177 | -0.039 to 0.393 |  |  |  |  |
|  |  |  |  |  |  |  |  |  |
| BREEDING SUCCESS |  |  |  |  |  |  |  |  |
| Bird type |  |  |  |  | 4.3 | 0.13 | 2 | 157.2 |
|  | Corvids | 9 | 1.093 | 0.474 to 1.713 |  |  |  |  |
|  | Small passerines | 20 | 0.435 | 0.042 to 0.828 |  |  |  |  |
|  | Birds of prey | 5 | 0.182 | -0.541 to 0.906 |  |  |  |  |
|  |  |  |  |  |  |  |  |  |

**References**

1. Borenstein M, Hedges LV, Higgins JPT, Rothstein HR: *Introduction to Meta-Analysis*. UK: John Wiley & Sons, Ltd; 2009:421.

2. Viechtbauer W: **Conducting Meta-Analyses in R with the metafor Package**. *J Stat Softw* 2010, **36**:1–47.
