# Supplementary material for: Reproductive responses of birds to experimental food supplementation: a meta-analysis
Source: Front Zool. 2014 Oct 31;11:80. doi: 10.1186/s12983-014-0080-y (PMC4222371; doi:10.1186/s12983-014-0080-y)
Supplement: Additional file 3: — List of all the publications included in the meta-analyses testing the effect of food supplementation on several reproductive parameters. [file 12983_2014_80_MOESM3_ESM.docx]

**Additional file 3**. **List of all the publications included in the meta-analyses testing the effect of food supplementation on several reproductive parameters.**

Data on the behaviour and life-history traits of the species were collated from original publications or other literature [1–4], and internet (<http://www.oiseaux.net/>)

BirdType: the “small passerine” category refers to passerines other than corvids; Diet: generalized diets (gener)/other diets (other); FoodAccess: level of accessibility to the feed; Broodedness: single-brooded/multi-brooded (i.e., more than two clutches per breeding season); Latitude: in decimal degrees; Elevation: in meters a.s.l.; Ntot: summed sample size of controls and food treatments (sample sizes refer to the number of nests or breeding pairs); Timing: timing of food supplementation: before and/or during egg-laying only (prelay/lay)/other timings (hatching to fledging or throughout the breeding season); BodyMass: body mass in grams; Parameter: laying date (LD), clutch size (CS), breeding success (BS); FoodLevel: relative index of food abundance in the environment (higher/lower than average).

**References**

1. Cramp S, Simmons KEL, Brooks DC, Collar NJ, Dunn E, Gillmor R, Hollom PAD, Hudson R, Nicholson EM, Ogilvie MA, Olney PJS, Roselaar CS, Voous KH, Wallace DIM, Wattel J, Wilson MG: *Handbook of the Birds of Europe, the Middle East and North Africa. The Birds of the Western Palearctic*. Oxford, UK: Oxford University Press; 1983.

2. Dunning JJ: *CRC Handbook of Avian Body Masses - Second Edition*. CRC Press; 2008.

3. Schoech SJ: **A reply to Dhondt: broodedness and latitude affect the response of reproductive timing of birds to food supplementation**. *J Ornithol* 2010, **151**:959–961.

4. Dhondt AA: **Broodedness, not latitude, affects the response of reproductive timing of birds to food supplementation**. *J Ornithol* 2010, **151**:955–957.

**3a**. List of all the publications included in the meta-analyses testing the effect of food supplementation on laying date, sorted by reference.

|  | **Reference** | **Species** | **BirdType** | **Diet** | **FoodAccess** | **Migratory** | **Broodedness** | **Latitude** | **Elevation** | **Response** | ***d*** | **Var(*d*)** | **Ntot** |
| --- | --- | --- | --- | --- | --- | --- | --- | --- | --- | --- | --- | --- | --- |
| 1 | Aparicio & Bonal, 2002 | *Falco naumanni* | bird of prey | other | high | yes | single | 39.33 | 628 | mean laying date: 1999 (fed vs. unfed) | 0.674 | 0.058 | 77 |
| 2 | Aparicio, 1994 | *Falco tinnunculus* | bird of prey | other | high | no | single | 40.13 | 1000 | mean laying date: 1990 (early fed) | 2.300 | 0.288 | 24 |
| 3 | Arnold, 1992 | *Xanthocephalus xanthocephalus* | small passerine | other | low | yes | single | 50.17 | 520 | mean laying date: 1989 | 0.328 | 0.012 | 351 |
| 4 | Arnold, 1994 | *Fulica americana* | wetland bird | gener | intermediate | yes | single | 50.17 | 520 | mean laying date: 1987-1989/1991 | 0.241 | 0.006 | 695 |
| 5 | Bolton *et al*., 1992 | *Larus fuscus* | seabird | gener | intermediate | no | single | 51.37 | 15 | mean laying date: 1989 (fish fed-experiment 1) | 0.000 | 0.077 | 55 |
| 6 | Bourgault *et al*., 2009 | *Cyanistes caeruleus* | small passerine | other | intermediate | no | single | 42.00 | 300 | mean laying date: all years (all sites) | 0.419 | 0.014 | 302 |
| 7 | Clamens & Insenmann, 1989 | *Cyanistes caeruleus* | small passerine | other | intermediate | no | single | 43.92 | 100 | mean laying date: 1986-1987 | 1.250 | 0.076 | 68 |
| 8 | Clamens & Insenmann, 1989 | *Parus major* | small passerine | other | intermediate | no | multi | 43.92 | 100 | mean laying date: 1986-1987 | 0.535 | 0.109 | 38 |
| 9 | Davies & Lundberg, 1985 | *Prunella modularis* | small passerine | other | low | yes | multi | 52.18 | 19 | mean laying date: 1982-1983 | 1.209 | 0.089 | 54 |
| 10 | De Neve *et al*., 2004 | *Pica pica* | corvid | gener | high | no | single | 37.30 | 1000 | mean laying date: 2000 | 0.321 | 0.028 | 208 |
| 11 | Dhindsa & Boag, 1990 | *Pica pica* | corvid | gener | high | no | single | 53.50 | 740 | mean laying date: 1987-1988 | 1.138 | 0.151 | 35 |
| 12 | Dijkstra *et al*., 1982 | *Falco tinnunculus* | bird of prey | other | high | no | single | 53.33 | 0 | mean laying date: 1978-1980 (nests occupied bef. 03.1982) | 1.125 | 0.130 | 38 |
| 13 | Harrison *et al*., 2010 | *Cyanistes caeruleus* | small passerine | other | low | no | single | 52.60 | 150 | mean laying date: 2006-2008 | 0.373 | 0.011 | 427 |
| 14 | Harrison *et al.*, 2010 | *Parus major* | small passerine | other | low | no | single | 52.60 | 150 | mean laying date: 2006-2008 | 0.706 | 0.018 | 268 |
| 15 | Hiom *et al*., 1991 | *Larus fuscus* | seabird | gener | high | yes | single | 51.33 | 20 | mean laying date: 1988-1989 (Skomer/Flat Holm) | 0.000 | 0.036 | 120 |
| 16 | Hochachka & Boag, 1987 | *Pica pica* | corvid | gener | intermediate | no | single | 53.55 | 670 | mean laying date: 1982-1983 (all habitats) | 0.778 | 0.063 | 69 |
| 17 | Högstedt, 1981 | *Pica pica* | corvid | gener | high | no | single | 55.40 | 670 | mean laying date: 1974-1975 | 0.436 | 0.082 | 53 |
| 18 | Hörnfeldt & Eklund, 1990 | *Aegolius funereus* | bird of prey | other | high | yes | single | 64.00 | 250 | mean laying date: 1985 | 0.688 | 0.056 | 76 |
| 19 | Kelly & Van Horne, 1997 | *Megaceryle alcyon* | other type | other | intermediate | yes | single | 40.58 | 1500 | mean laying date: 1993-1995 | 1.059 | 0.127 | 36 |
| 20 | Knight, 1988 | *Pica pica* | corvid | gener | intermediate | no | single | 47.00 | 325 | mean laying date: 1981 (all controls combined) | 0.682 | 0.137 | 23 |
| 21 | Komdeur, 1996 | *Copsychus sechellarum* | small passerine | other | high | no | multi | 4.58 | 50 | mean laying date: 1990 | 1.689 | 0.543 | 10 |
| 22 | Korpimäki & Wiehn, 1998 | *Falco tinnunculus* | bird of prey | other | high | yes | single | 63.00 | 40 | mean laying date: 1986-1988 | -0.061 | 0.096 | 42 |
| 23 | Källander & Karlsson, 1993 | *Sturnus vulgaris* | small passerine | gener | low | yes | single | 55.50 | 300 | mean laying date: 1982/1985/1990 | 2.339 | 0.077 | 96 |
| 24 | Källander, 1974 | *Parus major* | small passerine | other | low | no | multi | 55.50 | 300 | mean laying date: 1972-1973 | 1.818 | 0.050 | 119 |
| 25 | Martínez-Padilla *et al*., 2006 | *Falco tinnunculus* | bird of prey | other | high | no | single | 40.60 | 1300 | mean laying date: 2002 | -0.013 | 0.121 | 33 |
| 26 | Meijer *et al*., 1988 | *Falco tinnunculus* | bird of prey | other | high | no | single | 53.33 | 0 | mean laying date: 1978-1980/1985-1986 (early feeding) | 0.849 | 0.112 | 49 |
| 27 | Millon *et al*., 2008 | *Circus pygargus* | bird of prey | other | high | yes | single | 46.10 | 50 | mean laying date: all years (sites DS/R0) | 0.298 | 0.074 | 84 |
| 28 | Nakamura, 1995 | *Prunella collaris* | small passerine | other | intermediate | yes | multi | 36.10 | 2813 | mean laying date: 1986-1989 - young & old females | 1.033 | 0.065 | 70 |
| 29 | Nilsson, 1994/Nilsson & Svensson, 1993 | *Cyanistes caeruleus* | small passerine | other | intermediate | no | single | 55.67 | 80 | mean laying date: 1990-1991 | 0.775 | 0.031 | 229 |
| 30 | Pihlaja *et al*., 2006 | *Pica pica* | corvid | gener | high | no | single | 62.00 | 125 | mean laying date: 2002 | 0.154 | 0.255 | 16 |
| 31 | Poole, 1985 | *Pandion haliaetus* | bird of prey | other | high | yes | single | 40.53 | 15 | mean laying date: 1980 | 0.550 | 0.519 | 8 |
| 32 | Ramsay & Houston, 1998 | *Cyanistes caeruleus* | small passerine | other | high | no | single | 56.13 | 100 | mean laying date: 1995 (high protein treatment) | 0.438 | 0.102 | 40 |
| 33 | Reynolds *et al*., 2003 | *Aphelocoma coerulescens* | corvid | other | high | no | single | 27.17 | 53 | mean laying date: 2000-2001 (high fat/high protein treatment) | 1.692 | 0.085 | 76 |
| 34 | Robb *et al*., 2008 | *Cyanistes caeruleus* | small passerine | other | high | no | single | 54.33 | 50 | mean laying date: 2006 | 0.525 | 0.056 | 76 |
| 35 | Sanz & Moreno, 1995 | *Ficedula hypoleuca* | small passerine | other | high | yes | single | 40.80 | 1900 | mean laying date: 1993 | -0.044 | 0.133 | 30 |
| 36 | Scheuerlein & Gwinner, 2002 | *Saxicola torquatus* | small passerine | other | intermediate | no | multi | 3.25 | 2700 | mean laying date: 1996 | 2.081 | 0.343 | 18 |
| 37 | Schoech, 1996 | *Aphelocoma coerulescens* | corvid | other | high | no | single | 27.17 | 53 | mean laying date: 1993 | 2.456 | 0.225 | 38 |
| 38 | Smith *et al*., 1980 | *Melospiza melodia* | small passerine | other | low | no | multi | 48.63 | 0 | mean laying date: 1979 (known access to feeders) | 1.233 | 0.236 | 29 |
| 39 | Soler & Soler, 1996 | *Corvus monedula* | corvid | gener | high | no | single | 37.30 | 1000 | mean laying date: 1983 | 0.519 | 0.137 | 31 |
| 40 | Spottiswoode, 2009 | *Philetairus socius* | small passerine | other | intermediate | no | multi | 24.67 | 1200 | mean laying date: 2004 | 2.477 | 0.073 | 98 |
| 41 | Wimberger, 1988 | *Agelaius phoeniceus* | small passerine | gener | intermediate | yes | single | 47.00 | 325 | mean laying date: 1981 (Skeleton/Infinity) | 2.187 | 0.320 | 20 |

**References**

Aparicio JM, Bonal R: **Effects of food supplementation and habitat selection on timing of lesser kestrel breeding**. *Ecology* 2002, **83**:873–877.

Aparicio JM: **The seasonal decline in clutch size: an experiment with supplementary food in the kestrel, *Falco tinnunculus***. *Oikos* 1994, **71**:451–458.

Arnold TW: **Variation in laying date, clutch size, egg size, and egg composition of yellow-headed blackbirds (*Xanthocephalus xanthocephalus*): a supplemental feeding experiment**. *Can J Zool* 1992, **70**:1904–1911.

Arnold TW: **Effect of supplemental food on egg production in American coots**. *Auk* 1994, **111**:337–350.

Bolton M, Houston DC, Monaghan P: **Nutritional constraints on egg formation in the lesser black-backed gull: an experimental study**. *J Anim Ecol* 1992, **61**:521–532.

Bourgault P, Perret P, Lambrechts MM: **Food supplementation in distinct Corsican oak habitats and the timing of egg laying by Blue Tits**. *J F Ornithol* 2009, **80**:127–134.

Clamens A, Isenmann P: **Effect of supplemental food on the breeding of Blue and Great Tits in Mediterranean habitats**. *Ornis Scand* 1989, **20**:36–42.

Davies NB, Lundberg A: **The influence of food on time budget and timing of breeding of the Dunnock *Prunella modularis***. *Ibis* 1985, **127**:100–110.

Dhindsa MS, Boag DA: **The effect of food supplementation on the reproductive success of Black-billed Magpies Pica pica**. *Ibis* 1990, **132**:595–602.

De Neve L, Soler JJ, Soler M, Martín-Vivaldi M, Martínez JG: **Effects of a food supplementation experiment on reproductive investment and a post-mating sexually selected trait in magpies *Pica pica***. *J Avian Biol* 2004, **35**:246–251.

Dijkstra C, Vuursteen L, Daan S, Masman D: **Clutch size and laying date in the kestrel *Falco tinnunculus*: effect of supplementary food**. *Ibis* 1982, **124**:210–213.

Harrison TJE, Smith JA, Martin GR, Chamberlain DE, Bearhop S, Robb GN, Reynolds SJ: **Does food supplementation really enhance productivity of breeding birds?** *Oecologia* 2010, **164**:311–20.

Hiom L, Bolton M, Monaghan P, Worrall D: **Experimental evidence for food limitation of egg production in gulls**. *Ornis Scand* 1991, **22**:94–97.

Hochachka WM, Boag A: **Food shortage for breeding Black-billed Magpies (*Pica pica*): an experiment using supplemental food**. *Can J Zool* 1987, **65**:1270–1274.

Högstedt G: **Effect of additional food on reproductive success in the Magpie (*Pica pica*)**. *J Anim Ecol* 1981, **50**:219–229.

Hörnfeldt B, Eklund U: **The effect of food on laying date and clutch-size in Tengmalm´s Owl *Aegolius funereus***. *Ibis* 1990, **132**:395–406.

Kelly JF, Van Horne B: **Effects of food supplementation on the timing of nest initiation in Belted Kingfishers**. *Ecology* 1997, **78**:2504–2511.

Knight RL: **Effects of supplemental food on the breeding biology of the Black-Billed Magpie**. *Condor* 1988, **90**:956–958.

Komdeur J: **Breeding of the Seychelles Magpie Robin *Copsychus sechellarum* and implications for its conservation**. *Ibis* 1996, **138**:485–498.

Korpimäki E, Wiehn J: **Clutch size of kestrels : seasonal decline and experimental evidence for food limitation under fluctuating food conditions**. *Oikos* 1998, **83**:259–272.

Källander H, Karlsson J: **Supplemental food and laying date in the European starling**. *Condor* 1993, **95**:1031–1034.

Källander H: **Advancement of laying of great tits by the provision of food**. *Ibis* 974, **116**:365–367.

Martínez-Padilla J: **Prelaying maternal condition modifies the association between egg mass and T cell-mediated immunity in kestrels**. *Behav Ecol Sociobiol* 2006, **60**:510–515.

Meijer T, Daan S, Dijkstra C: **Female condition and reproduction: effects of food manipulation in free-living and captive kestrels**. *Ardea* 1988, **76**:141–154.

Millon A, Arroyo BE, Bretagnolle V: **Variable but predictable prey availability affects predator breeding success: natural versus experimental evidence**. *J Zool* 2008, **275**:349–358.

Nakamura M: **Effects of supplemental feeding and female age on timing of breeding in the Alpine Accentor *Prunella collaris***. *Ibis* 1995, **137**:56–63.

Nilsson J-Å: **Energetic bottle-necks during breeding and the reproductive cost of being too early**. *J Anim Ecol* 1994, **63**:200–208.

Nilsson J-Å, Svensson E: **Energy constraints and ultimate decisions during egg-laying in the blue tit**. *Ecology* 1993, **74**:244–251.

Pihlaja M, Siitari H, Alatalo RV: **Maternal antibodies in a wild altricial bird: effects on offspring immunity, growth and survival.** *J Anim Ecol* 2006, **75**:1154–1164.

Poole A: **Courtship feeding and osprey reproduction**. *Auk* 1985, **102**:479–492.

Ramsay SL, Houston DC: **The effect of dietary amino acid composition on egg production in blue tits**. *Proc R Soc B Biol Sci* 1998, **265**:1401–1405.

Reynolds SJ, Schoech SJ, Bowman R: **Nutritional quality of prebreeding diet influences breeding performance of the Florida scrub-jay.** *Oecologia* 2003, **134**:308–316.

Robb GN, McDonald RA, Chamberlain DE, Bearhop S: **Food for thought: supplementary feeding as a driver of ecological change in avian populations**. *Front Ecol Environ* 2008, **6**:476–484.

Sanz JJ, Moreno J: **Experimentally induced clutch size enlargements affect reproductive success in the Pied Flycatcher**. *Oecologia* 1995, **103**:358–364.

Scheuerlein A, Gwinner E: **Is food availability a circannual zeitgeber in tropical birds? A field experiment on stonechats in tropical Africa**. *J Biol Rhythms* 2002, **17**:171–180.

Schoech SJ: **The effect of supplemental food on body condition and the timing of reproduction in a cooperative breeder, the Florida scrub-jay**. *Condor* 1996, **98**:234–244.

Smith JNM, Montgomerie RD, Taitt MJ, Yom-Tov Y: **A winter feeding experiment on an island song sparow population**. *Oecologia* 1980, **47**:164–170.

Soler M, Soler JJ: **Effects of experimental food provisioning on reproduction in the jackdaw *Corvus monedula*, a semi-colonial species**. *Ibis* 1996, **138**:377–383.

Spottiswoode CN: **Fine-scale life-history variation in sociable weavers in relation to colony size.** *J Anim Ecol* 2009, **78**:504–12.

Wimberger PH: **Food supplement effects on breeding time and harem size in the red-winged blackbird (*Agelaius phoeniceus*)**. *Auk* 1988, **105**:799–802.

**3b**. List of all the publications included in the meta-analyses testing the effect of food supplementation on clutch size, sorted by reference.

|  | **Reference** | **Species** | **BirdType** | **Diet** | **FoodAccess** | **BodyMass** | **Latitude** | **Migratory** | **MaxClutchSize** | **Broodedness** | **FoodCaching** | **Response** | ***d*** | **Var(*d*)** | **Ntot** |
| --- | --- | --- | --- | --- | --- | --- | --- | --- | --- | --- | --- | --- | --- | --- | --- |
| 1 | Aparicio, 1994 | *Falco tinnunculus* | bird of prey | other | high | 204.0 | 40.13 | no | 6 | single | yes | mean clutch size: 1990 (early fed) | 1.106 | 0.203 | 24 |
| 2 | Arcese & Smith, 1988 | *Melospiza melodia* | small passerine | other | high | 32.5 | 48.63 | no | 5 | multi | no | mean clutch size: 1985 | 0.524 | 0.046 | 97 |
| 3 | Arnold, 1992 | *Xanthocephalus xanthocephalus* | small passerine | other | low | 72.0 | 50.17 | yes | 5 | single | no | mean clutch size: 1989 | 0.119 | 0.017 | 247 |
| 4 | Arnold, 1994 | *Fulica americana* | wetland bird | gener | intermediate | 726.0 | 50.17 | yes | 9 | single | no | mean clutch size: 1987-1989/1991 | 0.531 | 0.008 | 531 |
| 5 | Bolton *et al.*, 1992 | *Larus fuscus* | seabird | gener | intermediate | 825.0 | 51.37 | no | 3 | single | no | mean clutch size: 1989-1990 (fish fed) | 0.298 | 0.037 | 114 |
| 6 | Carlson, 1989 | *Lanius collurio* | small passerine | other | high | 34.0 | 59.00 | yes | 6 | multi | yes | mean clutch size: 1984/1986 | 1.826 | 0.258 | 22 |
| 7 | Castro *et al*., 2003 | *Notiomystis cincta* | small passerine | other | intermediate | 32.5 | 38.07 | no | 5 | multi | no | mean clutch size: 1995-1997 | 0.748 | 0.122 | 35 |
| 8 | Clamens & Isenmann, 1989 | *Cyanistes caeruleus* | small passerine | other | intermediate | 10.5 | 43.92 | no | 13 | single | no | mean clutch size: 1986-1987 | -0.313 | 0.068 | 67 |
| 9 | Clamens & Isenmann, 1989 | *Parus major* | small passerine | other | intermediate | 18.5 | 43.92 | no | 18 | multi | no | mean clutch size: 1986-1987 | -0.790 | 0.127 | 34 |
| 10 | Clifford & Anderson, 2001 | *Sula granti* | seabird | other | high | 1900.0 | 1.33 | yes | 2 | single | no | mean clutch size: 1996-1997 | 0.464 | 0.042 | 99 |
| 11 | Davies & Lundberg, 1985 | *Prunella modularis* | small passerine | other | low | 20.0 | 52.18 | no | 6 | multi | no | mean clutch size: 1982-1983 (first clutches) | -0.133 | 0.032 | 126 |
| 12 | De Neve *et al*., 2004 | *Pica pica* | corvid | gener | high | 225.0 | 37.30 | no | 9 | single | yes | mean clutch size: 2000 | 0.079 | 0.034 | 166 |
| 13 | Dhindsa & Boag, 1990 | *Pica pica* | corvid | gener | high | 225.0 | 53.50 | no | 9 | single | yes | mean clutch size: 1987-1988 | 0.666 | 0.150 | 33 |
| 14 | Dijkstra *et al.*, 1982 | *Falco tinnunculus* | bird of prey | other | high | 204.0 | 53.33 | no | 6 | single | yes | mean clutch size: 1978-1980 (nests occupied bef. 03.1982) | 0.953 | 0.135 | 35 |
| 15 | Eikenaar *et al*., 2003 | *Acrocephalus australis* | small passerine | other | high | 18.0 | 38.03 | yes | 6 | multi | no | mean clutch size: 2000-2001 | -0.110 | 0.079 | 68 |
| 16 | Ewald & Rohwer, 1982 | *Agelaius phoeniceus* | small passerine | gener | intermediate | 55.0 | 47.17 | yes | 5 | single | no | mean clutch size: 1977-1978 | -0.200 | 0.008 | 489 |
| 17 | Gill & Hatch, 2002 | *Rissa tridactyla* | seabird | other | high | 400.0 | 55.70 | yes | 2 | single | no | mean clutch size: 1996-1997 (fed all seasons) | 0.243 | 0.029 | 168 |
| 18 | Harrison *et al.*, 2010 | *Cyanistes caeruleus* | small passerine | other | low | 10.5 | 52.60 | no | 13 | single | no | mean clutch size: 2006-2008 | -0.370 | 0.012 | 379 |
| 19 | Harrison *et al.*, 2010 | *Parus major* | small passerine | other | low | 18.5 | 52.60 | no | 18 | single | no | mean clutch size: 2006-2008 | -0.464 | 0.017 | 259 |
| 20 | Hill, 1988 | *Fulica americana* | wetland bird | gener | intermediate | 726.0 | 47.40 | no | 9 | single | no | mean clutch size: 1982 | -0.094 | 0.584 | 7 |
| 21 | Hillström, 1995 | *Ficedula hypoleuca* | small passerine | other | high | 12.5 | 59.83 | yes | 8 | single | no | mean clutch size: 1986/1989 (low food ration) | 0.137 | 0.106 | 57 |
| 22 | Hiom *et al*., 1991 | *Larus fuscus* | seabird | gener | high | 825.0 | 51.33 | no | 3 | single | no | mean clutch size: 1988-1989 (Skomer/Flat Holm) | 0.324 | 0.036 | 120 |
| 23 | Hochachka & Boag, 1986 | *Pica pica* | corvid | gener | intermediate | 225.0 | 53.55 | no | 9 | single | yes | mean clutch size: all years (all habitats) | 0.374 | 0.052 | 85 |
| 24 | Högstedt, 1981 | *Pica pica* | corvid | gener | high | 225.0 | 55.67 | no | 9 | single | yes | mean clutch size: 1974-1975 | 0.482 | 0.091 | 47 |
| 25 | Hörnfeldt & Eklund, 1990 | *Aegolius funereus* | bird of prey | other | high | 150.0 | 64.00 | yes | 7 | single | yes | mean clutch size: 1985 | 0.848 | 0.055 | 80 |
| 26 | Korpimäki & Wiehn, 1998 | *Falco tinnunculus* | bird of prey | other | high | 204.0 | 63.00 | yes | 6 | single | yes | mean clutch size: 1986-1988 | 1.145 | 0.111 | 42 |
| 27 | Källander & Karlsson, 1993 | *Sturnus vulgaris* | small passerine | gener | low | 82.0 | 55.50 | yes | 5 | single | no | mean clutch size: 1982/1985/1990 | 0.116 | 0.049 | 93 |
| 28 | Mackintosh & Briskie, 2005 | *Petroica australis australis* | small passerine | other | high | 36.0 | 41.00 | no | 3 | multi | yes | mean clutch size: 2001-2002 | 1.241 | 0.107 | 45 |
| 29 | Martínez-Padilla *et al*., 2006 | *Falco tinnunculus* | bird of prey | other | high | 204.0 | 40.66 | no | 6 | single | yes | mean clutch size: 2002 | 0.051 | 0.121 | 33 |
| 30 | Meijer *et al.*, 1988 | *Falco tinnunculus* | bird of prey | other | high | 204.0 | 53.33 | no | 6 | single | yes | mean clutch size: all years (early feeding) | 0.290 | 0.121 | 45 |
| 31 | Millon *et al*., 2008 | *Circus pygargus* | bird of prey | other | high | 338.0 | 46.10 | yes | 5 | single | no | mean clutch size: all years (sites DS/R0) | 0.529 | 0.037 | 173 |
| 32 | Nilsson, 1991 | *Poecile palustris* | small passerine | other | intermediate | 10.5 | 55.67 | no | 10 | single | yes | mean clutch size: 1988-1989 | 0.996 | 0.098 | 46 |
| 33 | Nilsson, 1994 | *Cyanistes caeruleus* | small passerine | other | intermediate | 10.5 | 55.67 | no | 13 | single | no | mean clutch size: 1991 | -0.012 | 0.080 | 88 |
| 34 | Pihlaja *et al.*, 2006 | *Pica pica* | corvid | gener | high | 225.0 | 62.00 | no | 9 | single | yes | mean clutch size: 2002 | -0.165 | 0.212 | 19 |
| 35 | Poole, 1985 | *Pandion haliaetus* | bird of prey | other | high | 1800.0 | 40.53 | yes | 3 | single | no | mean clutch size: 1980 | -1.491 | 0.639 | 8 |
| 36 | Ramsay & Houston, 1998 | *Cyanistes caeruleus* | small passerine | other | high | 10.5 | 56.13 | no | 13 | single | no | mean clutch size: 1995 (high protein treatment) | 0.780 | 0.108 | 40 |
| 37 | Reynolds *et al.*, 2003 | *Aphelocoma coerulescens* | corvid | other | high | 75.0 | 27.17 | no | 4 | single | yes | mean clutch size: 2000-2001 (high fat/high protein treatment) | 0.096 | 0.066 | 76 |
| 38 | Sanz & Moreno, 1995 | *Ficedula hypoleuca* | small passerine | other | high | 12.5 | 40.80 | yes | 8 | single | no | mean clutch size: 1993 | 0.740 | 0.143 | 30 |
| 39 | Schoech *et al*., 2008 | *Aphelocoma coerulescens* | corvid | other | high | 75.0 | 27.17 | no | 4 | single | yes | mean clutch size: 2000-2006 | 1.010 | 0.016 | 311 |
| 40 | Schoech, 1996 | *Aphelocoma coerulescens* | corvid | other | high | 75.0 | 27.17 | no | 4 | single | yes | mean clutch size: 1993 | 0.619 | 0.148 | 40 |
| 41 | Smith *et al*., 1980 | *Melospiza melodia* | small passerine | other | low | 32.5 | 48.63 | no | 5 | multi | no | mean clutch size: 1979 (known access to feeders) | -1.397 | 0.244 | 29 |
| 42 | Soler & Soler, 1996 | *Corvus monedula* | corvid | gener | high | 245.0 | 37.30 | no | 7 | single | no | mean clutch size: 1983 | 0.968 | 0.148 | 31 |
| 43 | Spottiswoode, 2009 | *Philetairus socius* | small passerine | other | intermediate | 27.0 | 24.67 | no | 6 | multi | no | mean clutch size: 2004 | -0.454 | 0.043 | 96 |
| 44 | von Brömssen & Jansson, 1980 | *Lophophanes cristatus* | small passerine | other | intermediate | 11.0 | 57.67 | no | 8 | single | yes | mean clutch size: 1976-1977 | 0.107 | 0.159 | 38 |
| 45 | von Brömssen & Jansson, 1980 | *Poecile montanus* | small passerine | other | intermediate | 10.5 | 57.67 | no | 9 | single | yes | mean clutch size: 1976-1977 | 0.044 | 0.093 | 44 |
| 46 | Zanette *et al*., 2006 | *Melospiza melodia* | small passerine | other | intermediate | 32.5 | 48.33 | no | 5 | multi | no | mean clutch size: 2000-2002 | 0.476 | 0.018 | 231 |

**References**

Aparicio JM: **The seasonal decline in clutch size: an experiment with supplementary food in the kestrel, *Falco tinnunculus****.* *Oikos* 1994, **71**:451–458.

Arcese P, Smith JNM: **Effects of population density and supplemental food on reproduction in song sparrows**. *J Anim Ecol* 1988, **57**:119–136.

Arnold TW: **Variation in laying date, clutch size, egg size, and egg composition of yellow-headed blackbirds (*Xanthocephalus xanthocephalus*): a supplemental feeding experiment**. *Can J Zool* 1992, **70**:1904–1911.

Arnold TW: **Effect of supplemental food on egg production in American coots**. *Auk* 1994, **111**:337–350.

Bolton M, Houston DC, Monaghan P: **Nutritional constraints on egg formation in the lesser black-backed gull: an experimental study**. *J Anim Ecol* 1992, **61**:521–532.

von Brömssen A, Jansson C: **Effects of food addition to Willow Tit *Parus montanus* and Crested Tit *P. cristatus* at the time of breeding**. *Ornis Scand* 1980, **11**:173–178.

Carlson A: **Courtship feeding and clutch size in Red-Backed Shrikes (*Lanius collurio*)**. *Am Nat* 1989, **133**:454–457.

Castro I, Brunton DH, Mason KM, Ebert B, Griffiths R: **Life history traits and food supplementation affect productivity in a translocated population of the endangered Hihi (Stitchbird, *Notiomystis cincta*)**. *Biol Conserv* 2003, **114**:271–280.

Clamens A, Isenmann P: **Effect of supplemental food on the breeding of Blue and Great Tits in Mediterranean habitats**. *Ornis Scand* 1989, **20**:36–42.

Clifford LD, Anderson DJ: **Food limitation explains most clutch size variation in the Nazca booby**. *J Anim Ecol* 2001, **70**:539–545.

Davies NB, Lundberg A: **The influence of food on time budget and timing of breeding of the Dunnock *Prunella modularis***. *Ibis 1*985, **127**:100–110.

De Neve L, Soler JJ, Soler M, Martín-Vivaldi M, Martínez JG: **Effects of a food supplementation experiment on reproductive investment and a post-mating sexually selected trait in magpies *Pica pica***. *J Avian Biol* 2004, **35**:246–251.

Dhindsa MS, Boag DA: **The effect of food supplementation on the reproductive success of Black-billed Magpies *Pica pica***. *Ibis*  1990, **132**:595–602.

Dijkstra C, Vuursteen L, Daan S, Masman D: **Clutch size and laying date in the kestrel *Falco tinnunculus*: effect of supplementary food**. *Ibis* 1982, **124**:210–213.

Eikenaar C, Berg ML, Komdeur J: **Experimental evidence for the influence of food availability on incubation attendance and hatching asynchrony in the Australian reed warbler *Acrocephalus australis***. *J Avian Biol* 2003, **34**:419–427.

Ewald PW, Rohwer S: **Effects of supplemental feeding on timing of breeding, clutch-size and polygyny in red-winged blackbirds *Agelaius phoeniceus***. *J Anim Ecol* 1982, **51**:429–450.

Gill VA, Hatch SA: **Components of productivity in black-legged kittiwakes *Rissa tridactyla*: response to supplemental feeding**. *J Avian Biol* 2002, **33**:113–126.

Harrison TJE, Smith JA, Martin GR, Chamberlain DE, Bearhop S, Robb GN, Reynolds SJ: **Does food supplementation really enhance productivity of breeding birds?** *Oecologia* 2010, **164**:311–20.

Hill WL: **The effect of food abundance on the reproductive patterns of coots**. *Condor* 1988, **90**:324–331.

Hillström L: **Body mass reductionin during reproduction in the Pied Flycatcher *Ficedula hypoleuca*: physiological stress or adaptation for lowered costs of locomotion?** *Funct Ecol* 1995, **9**:807–817.

Hiom L, Bolton M, Monaghan P, Worrall D: **Experimental evidence for food limitation of egg production in gulls**. *Ornis Scand* 1991, **22**:94–97.

Hochachka WM, Boag A: **Food shortage for breeding Black-billed Magpies (*Pica pica*): an experiment using supplemental food**. *Can J Zool* 1987, **65**:1270–1274.

Högstedt G: **Effect of additional food on reproductive success in the Magpie (*Pica pica*)**. *J Anim Ecol* 1981, **50**:219–229.

Källander H, Karlsson J: **Supplemental food and laying date in the European starling**. *Condor* 1993, **95**:1031–1034.

Mackintosh M, Briskie J: **High levels of hatching failure in an insular population of the South Island robin: a consequence of food limitation?** *Biol Conserv* 2005, **122**:409–416.

Martínez-Padilla J: **Prelaying maternal condition modifies the association between egg mass and T cell-mediated immunity in kestrels**. *Behav Ecol Sociobiol* 2006, **60**:510–515.

Meijer T, Daan S, Dijkstra C: **Female condition and reproduction: effects of food manipulation in free-living and captive kestrels**. *Ardea* 1988, **76**:141–154.

Millon A, Arroyo BE, Bretagnolle V: **Variable but predictable prey availability affects predator breeding success: natural versus experimental evidence**. *J Zool* 2008, **275**:349–358.

Nilsson J-Å: **Clutch size determination in the Marsh tit (*Parus palustris*)**. *Ecology* 1991, **72**:1757–1762.

Nilsson J-Å: **Energetic bottle-necks during breeding and the reproductive cost of being too early**. *J Anim Ecol* 1994, **63**:200–208.

Pihlaja M, Siitari H, Alatalo RV: **Maternal antibodies in a wild altricial bird: effects on offspring immunity, growth and survival.** *J Anim Ecol* 2006, **75**:1154–1164.

Poole A: **Courtship feeding and osprey reproduction**. *Auk* 1985, **102**:479–492.

Ramsay SL, Houston DC: **The effect of dietary amino acid composition on egg production in blue tits**. *Proc R Soc B Biol Sci* 1998, **265**:1401–1405.

Reynolds SJ, Schoech SJ, Bowman R: **Nutritional quality of prebreeding diet influences breeding performance of the Florida scrub-jay.** *Oecologia* 2003, **134**:308–316.

Sanz JJ, Moreno J: **Experimentally induced clutch size enlargements affect reproductive success in the Pied Flycatcher**. *Oecologia* 1995, **103**:358–364.

Schoech SJ, Bridge ES, Boughton RK, Reynolds SJ, Atwell JW, Bowman R: **Food supplementation: A tool to increase reproductive output? A case study in the threatened Florida Scrub-Jay**. *Biol Conserv* 2008, **141**:162–173.

Schoech SJ: **The effect of supplemental food on body condition and the timing of reproduction in a cooperative breeder, the Florida scrub-jay**. *Condor* 1996, **98**:234–244.

Smith JNM, Montgomerie RD, Taitt MJ, Yom-Tov Y: **A winter feeding experiment on an island song sparow population**. *Oecologia* 1980, **47**:164–170.

Soler M, Soler JJ: **Effects of experimental food provisioning on reproduction in the jackdaw *Corvus monedula*, a semi-colonial species**. *Ibis* 1996, **138**:377–383.

Spottiswoode CN: **Fine-scale life-history variation in sociable weavers in relation to colony size.** *J Anim Ecol* 2009, **78**:504–12.

Zanette L, Clinchy M, Smith JNM: **Food and predators affect egg production in song sparrows.** *Ecology* 2006, **87**:2459–67.

**3c**. List of all the publications included in the meta-analyses testing the effect of food supplementation on breeding success, sorted by reference.

|  | **Reference** | **Species** | **BirdType** | **BodyMass** | **Diet** | **FoodCaching** | **FoodAccess** | **Timing** | **Response** | ***d*** | **Var(*d*)** | **Ntot** |
| --- | --- | --- | --- | --- | --- | --- | --- | --- | --- | --- | --- | --- |
| 1 | Bolton *et al*., 1992 | *Larus fuscus* | seabird | 825.0 | gener | no | intermediate | prelay/lay | mean breeding success: 1990 (egg fed) | 0.103 | 0.073 | 55 |
| 2 | Brinkhof & Cavé, 1997 | *Fulica atra* | wetland bird | 730.0 | gener | no | intermediate | other | mean breeding success: 1990-1991 (all hatching dates) | 1.596 | 0.075 | 70 |
| 3 | Brömssen & Jansson, 1980 | *Lophophanes cristatus* | small passerine | 11.0 | other | yes | intermediate | prelay/lay | mean breeding success: 1976-1977 | -0.076 | 0.233 | 35 |
| 4 | Brömssen & Jansson, 1980 | *Poecile montanus* | small passerine | 10.5 | other | yes | intermediate | prelay/lay | mean breeding success: 1976-1977 | 0.051 | 0.120 | 46 |
| 5 | Byholm & Kekkonen, 2008 | *Accipiter gentilis* | bird of prey | 988.0 | other | yes | high | other | mean breeding success: 2004-2005 (forest/bog) | 0.042 | 0.047 | 87 |
| 6 | Castro *et al.*, 2003 | *Notiomystis cincta* | small passerine | 30.0 | other | no | intermediate | other | mean breeding success: 1995-1997 | 0.819 | 0.126 | 36 |
| 7 | Clamens & Insenmann, 1989 | *Cyanestes caeruleus* | small passerine | 10.5 | other | no | intermediate | other | mean breeding success: 1986-1987 | 0.849 | 0.092 | 52 |
| 8 | Clamens & Insenmann, 1989 | *Parus major* | small passerine | 18.5 | other | no | intermediate | other | mean breeding success: 1986-1987 | -0.245 | 0.141 | 29 |
| 9 | Davies & Lundberg, 1985 | *Prunella modularis* | small passerine | 20.0 | other | no | low | other | mean breeding success: 1982-1983 (all completed clutches) | -0.255 | 0.032 | 126 |
| 10 | Davis *et al.*, 2005 | *Stercorarius parasiticus* | seabird | 450.0 | gener | no | high | other | mean breeding success: 2001 | 0.669 | 0.080 | 53 |
| 11 | Dhindsa & Boag, 1990 | *Pica pica* | corvid | 225.0 | gener | yes | high | other | mean breeding success: 1987-1988 | 1.412 | 0.182 | 28 |
| 12 | Gill & Hatch, 2002 | *Rissa tridactyla* | seabird | 400.0 | other | no | high | other | mean breeding success: 1996-1997 (fed to all seasons) | 0.872 | 0.038 | 128 |
| 13 | Granbom & Smith, 2006 | *Sturnus vulgaris* | small passerine | 82.0 | gener | no | high | other | mean breeding success: 1998-1999 (high/low quality habitats) | 0.397 | 0.041 | 99 |
| 14 | Hillstrom, 1995 | *Ficedula hypoleuca* | small passerine | 12.5 | other | no | high | other | mean breeding success: 1986/1989 (low food ration) | 0.137 | 0.109 | 52 |
| 15 | Hochachka & Boag, 1986 | *Pica pica* | corvid | 225.0 | gener | yes | intermediate | other | mean breeding success: 1982-1983 (urban habitat) | 0.359 | 0.117 | 43 |
| 16 | Hochachka & Boag, 1986 | *Pica pica* | corvid | 225.0 | gener | yes | intermediate | other | mean breeding success: 1982-1983 (farm) | 0.507 | 0.244 | 17 |
| 17 | Hochachka & Boag, 1986 | *Pica pica* | corvid | 225.0 | gener | yes | intermediate | other | mean breeding success: 1982-1983 (river valley) | 0.649 | 0.171 | 26 |
| 18 | Högstedt, 1981 | *Pica pica* | corvid | 225.0 | gener | yes | high | other | mean breeding success: 1974-1975 | 0.863 | 0.101 | 46 |
| 19 | Komdeur, 1996 | *Copsychus sechellarum* | small passerine | 71.0 | other | no | high | other | mean breeding success: 1990 | 1.394 | 0.497 | 10 |
| 20 | Källander & Karlsson, 1993 | *Sturnus vulgaris* | small passerine | 82.0 | gener | no | low | prelay/lay | mean breeding success: 1982/1990 | -0.479 | 0.066 | 72 |
| 21 | Martinez-Padilla *et al*., 2006 | *Falco tinnunculus* | bird of prey | 204.0 | other | yes | high | prelay/lay | mean breeding success: 2002 | 0.539 | 0.126 | 33 |
| 22 | Million *et al.*, 2008 | *Circus pygargus* | bird of prey | 338.0 | other | no | high | prelay/lay | mean breeding success: all years (sites DS/RO) | 0.021 | 0.060 | 122 |
| 23 | Nagy & Holmes, 1995 | *Dendroica caerulescens* | small passerine | 18.5 | other | no | high | other | mean breeding success: 2000 | 0.470 | 0.249 | 17 |
| 24 | Nilsson, 1994 | *Cyanestes caeruleus* | small passerine | 10.5 | other | no | intermediate | prelay/lay | mean breeding success: 1991 | -0.473 | 0.170 | 48 |
| 25 | Preston & Rotenberry, 2006 | *Chamaea fasciata* | small passerine | 15.0 | other | no | intermediate | other | mean breeding success: 2001 | 0.601 | 0.103 | 41 |
| 26 | Ramsay & Houston, 1998 | *Cyanestes caeruleus* | small passerine | 10.5 | other | no | high | prelay/lay | mean breeding success: 1995 (high protein treatment) | 0.339 | 0.180 | 24 |
| 27 | Reynolds *et al*., 2003 | *Aphelocoma coerulescens* | corvid | 75.0 | gener | yes | high | other | mean breeding success: 2001-2002 | 1.636 | 0.107 | 55 |
| 28 | Richner, 1992 | *Corvus corone* | corvid | 510.0 | gener | no | high | other | mean breeding success: 1986-1989 | 1.837 | 0.121 | 95 |
| 29 | Robb *et al*., 2008 | *Cyanestes caeruleus* | small passerine | 10.5 | other | no | intermediate | prelay/lay | mean breeding success: 2006 | 0.468 | 0.056 | 76 |
| 30 | Sanz & Moreno, 1995 | *Ficedula hypoleuca* | small passerine | 12.5 | other | no | high | prelay/lay | mean breeding success: 1993 (excluding deserted nests) | -0.945 | 0.259 | 18 |
| 31 | Schoech *et al*., 2008 | *Aphelocoma coerulescens* | corvid | 75.0 | gener | yes | high | prelay/lay | mean breeding success: 2000-2006 | 0.406 | 0.014 | 311 |
| 32 | Smith *et al*., 1980 | *Melospiza melodia* | small passerine | 32.5 | other | no | low | prelay/lay | mean breeding success: 1979 (known access to feeders) | -0.326 | 0.212 | 29 |
| 33 | Soler & Soler, 1996 | *Corvus monedula* | corvid | 245.0 | gener | no | high | other | mean breeding success: 1983 | 1.228 | 0.173 | 28 |
| 34 | Wellicome *et al*., 1997 | *Athene cunicularia* | bird of prey | 192.0 | other | yes | high | other | mean breeding success: 1992-1993/1996 | 4.579 | 0.274 | 53 |
| 35 | Verboven *et al*., 2001 | *Parus major* | small passerine | 18.5 | other | no | high | other | mean breeding success: 1987-1990 | 1.021 | 0.039 | 161 |
| 36 | Verhulst, 1994 | *Ficedula hypoleuca* | small passerine | 12.5 | other | no | high | other | mean breeding success: 1987 | 0.000 | 0.150 | 27 |
| 37 | Wiehn & Korpimäki, 1997 | *Falco tinnunculus* | bird of prey | 204.0 | other | yes | high | other | mean breeding success: 1992-1993/1995 | 0.321 | 0.056 | 72 |
| 38 | Zanette *et al*., 2006 | *Melospiza melodia* | small passerine | 32.5 | other | no | high | other | mean breeding success: 2000-2002 | 0.479 | 0.038 | 112 |

**References**

Bolton M, Houston DC, Monaghan P: **Nutritional constraints on egg formation in the lesser black-backed gull: an experimental study**. *J Anim Ecol* 1992, **61**:521–532.

Brinkhof MWG, Cave a. J: **Food supply and seasonal variation in breeding success: an experiment in the European coot**. *Proc R Soc B Biol Sci* 1997, **264**:291–296.

von Bromssen A, Jansson C: **Effects of food addition to Willow Tit *Parus montanus* and Crested Tit *P. cristatus* at the time of breeding**. *Ornis Scand* 1980, **11**:173–178.

Byholm P, Kekkonen M: **Food regulates reproduction differently in different habitats: experimental evidence in the Goshawk.** *Ecology* 2008, **89**:1696–702.

Castro I, Brunton DH, Mason KM, Ebert B, Griffiths R: **Life history traits and food supplementation affect productivity in a translocated population of the endangered Hihi (Stitchbird, *Notiomystis cincta*)**. *Biol Conserv* 2003, **114**:271–280.

Clamens A, Isenmann P: **Effect of supplemental food on the breeding of Blue and Great Tits in Mediterranean habitats**. *Ornis Scand* 1989, **20**:36–42.

Davies NB, Lundberg A: **The influence of food on time budget and timing of breeding of the Dunnock *Prunella modularis***. *Ibis* 1985, **127**:100–110.

Davis SE, Nager RG, Furness RW: **Food availability affects adult survival as well as breeding success of parasitic jaegers**. *Ecology* 2005, **86**:1047–1056.

Dhindsa MS, Boag DA: **The effect of food supplementation on the reproductive success of Black-billed Magpies *Pica pica***. *Ibis* 1990, **132**:595–602.

Gill VA, Hatch SA: **Components of productivity in black-legged kittiwakes *Rissa tridactyla*: response to supplemental feeding**. *J Avian Biol* 2002, **33**:113–126.

Granbom M, Smith HG: **Food limitation during breeding in a heterogeneous landscape**. *Auk* 2006, **123**:97.

Hillstrom L: **Body mass reductionin during reproduction in the Pied Flycatcher *Ficedula hypoleuca*: physiological stress or adaptation for lowered costs of locomotion?** *Funct Ecol* 1995, **9**:807–817.

Hochachka WM, Boag A: **Food shortage for breeding Black-billed Magpies (*Pica pica*): an experiment using supplemental food**. *Can J Zool* 1987, **65**:1270–1274.

Högstedt G: **Effect of additional food on reproductive success in the Magpie (*Pica pica*)**. *J Anim Ecol* 1981, **50**:219–229.

Komdeur J: **Breeding of the Seychelles Magpie Robin *Copsychus sechellarum* and implications for its conservation**. *Ibis* 1996, **138**:485–498.

Källander H, Karlsson J: **Supplemental food and laying date in the European starling**. *Condor* 1993, **95**:1031–1034.

Martínez-Padilla J: **Prelaying maternal condition modifies the association between egg mass and T cell-mediated immunity in kestrels**. *Behav Ecol Sociobiol* 2006, **60**:510–515.

Millon A, Arroyo BE, Bretagnolle V: **Variable but predictable prey availability affects predator breeding success: natural versus experimental evidence**. *J Zool* 2008, **275**:349–358.

Nagy LR, Holmes RT: **Food limits annual fecundity of a migratory songbird: an experimental study**. *Ecology* 2005, **86**:675–681.

Nilsson J-Å, Svensson E: **Energy constraints and ultimate decisions during egg-laying in the blue tit**. *Ecology* 1993, **74**:244–251.

Nilsson J-Å: **Energetic bottle-necks during breeding and the reproductive cost of being too early**. *J Anim Ecol* 1994, **63**:200–208.

Preston KL, Rotenberry JT: **Independent effects of food and predator-mediated processes on annual fecundity in a songbird.** *Ecology* 2006, **87**:160–168.

Ramsay SL, Houston DC: **The effect of dietary amino acid composition on egg production in blue tits**. *Proc R Soc B Biol Sci* 1998, **265**:1401–1405.

Reynolds SJ, Schoech SJ, Bowman R: **Nutritional quality of prebreeding diet influences breeding performance of the Florida scrub-jay.** *Oecologia* 2003, **134**:308–316.

Richner H: **The effect of extra food on fitness in breeding carrion crows**. *Ecology* 1992, **73**:330–335.

Robb GN, McDonald RA, Chamberlain DE, Bearhop S: **Food for thought: supplementary feeding as a driver of ecological change in avian populations**. *Front Ecol Environ* 2008, **6**:476–484.

Sanz JJ, Moreno J: **Experimentally induced clutch size enlargements affect reproductive success in the Pied Flycatcher**. *Oecologia* 1995, **103**:358–364.

Schoech SJ, Bridge ES, Boughton RK, Reynolds SJ, Atwell JW, Bowman R: **Food supplementation: A tool to increase reproductive output? A case study in the threatened Florida Scrub-Jay**. *Biol Conserv* 2008, **141**:162–173.

Smith JNM, Montgomerie RD, Taitt MJ, Yom-Tov Y: **A winter feeding experiment on an island song sparow population**. *Oecologia* 1980, **47**:164–170.

Soler M, Soler JJ: **Effects of experimental food provisioning on reproduction in the jackdaw *Corvus monedula*, a semi-colonial species**. *Ibis* 1996, **138**:377–383.

Wellicome TI, Holroyd GL, Scalise K, Wiltse ER: **The effects of predator exclusion and food supplementation on Burrowing Owl (*Speotyto cunicularia*) population change in Saskatchewan**. In *Biology and conservation of owls of the Northern Hemisphere: 2nd International symposium*. Edited by Duncan JR, Johnson DH, Nicholls TH. St. Paul, MN: U.S. Dept. of Agriculture, Forest Service, North Central Forest Experiment Station; 1997:487–497.

Verboven N, Tinbergen JM, Verhulst S: **Food, reproductive success and multiple breeding in the great tit *Parus major***. *Ardea* 2001, **89**:387–406.

Verhulst S: **Supplementary food in the nestling phase affects reproductive success in pied flycatchers (*Ficedula hypoleuca*)**. *Auk* 1994, **111**:714–716.

Wiehn J, Korpimäki E: **Food limitation on brood size: experimental evidence in the Eurasian kestrel**. *Ecology* 1997, **78**:2043–2050.

Zanette L, Clinchy M, Smith JNM: **Combined food and predator effects on songbird nest survival and annual reproductive success: results from a bi-factorial experiment.** *Oecologia* 2006, **147**:632–40.

**3d**. List of all the publications included in the meta-analyses testing the effect of food supplementation on egg size, hatching success, brood size and chick body mass, sorted by reference.

|  | **Reference** | **Species** | **BirdType** | **Parameter** | **Response** | ***d*** | **Var(*d*)** | **Ntot** |
| --- | --- | --- | --- | --- | --- | --- | --- | --- |
| 1 | Clinchy *et al*. 2004 | *Melospiza melodia* | small passerine | brood size | mean brood size: 2002 | 0.471 | 0.187 | 22 |
| 2 | Hillstrom, 1995 | *Ficedula hypoleuca* | small passerine | brood size | mean brood size: 1986/1989 (high food ration) | 0.373 | 0.109 | 53 |
| 3 | Martinez-Padilla *et al*., 2006 | *Falco tinnunculus* | bird of prey | brood size | mean brood size: 2002 | 0.370 | 0.123 | 33 |
| 4 | Ramsay & Houston, 1998 | *Cyanistes caeruleus* | small passerine | brood size | mean brood size: 1995 (7 days old - high protein treatment) | 0.339 | 0.180 | 24 |
| 5 | Sanz & Moreno, 1995 | *Ficedula hypoleuca* | small passerine | brood size | mean brood size: 1993 (excluding deserted nests) | -0.733 | 0.249 | 18 |
| 1 | Arcese & Smith, 1988 | *Melospiza melodia* | small passerine | chick mass | mean nestling mass (age 6 day old) | 0.553 | 0.039 | 108 |
| 2 | Bolton *et al*., 1992 | *Larus fuscus* | seabird | chick mass | mean chick growth rate: 1990 (chicks a,b,c - fish fed) | -0.041 | 0.156 | 28 |
| 3 | Davies & Lundberg, 1985 | *Prunella modularis* | small passerine | chick mass | mean brood weight: 1982-1983 | -0.471 | 0.045 | 93 |
| 4 | Dhindsa & Boag, 1990 | *Pica pica* | corvid | chick mass | mean nestling mass: 1987-1988 (age 20 days old) | 2.752 | 0.342 | 23 |
| 5 | Garcia *et al*., 1993 | *Sialia currucoides* | small passerine | chick mass | mean chick growth rate: 1991 (age 18 days old – large food supplement) | 2.075 | 0.222 | 32 |
| 6 | Harris, 1978 | *Fratercula arctica* | small passerine | chick mass | mean fledging mass: 1975 - St Kilda/Isle of May | 0.492 | 0.102 | 113 |
| 7 | Hipkiss *et al*., 2002 | *Aegolius funereus* | bird of prey | chick mass | mean fledgling mass: 1998-1999 (males & females) | 0.952 | 0.148 | 34 |
| 8 | Högstedt, 1981 | *Pica pica* | corvid | chick mass | mean chick mass: 1974-1975 | 0.095 | 0.138 | 29 |
| 9 | Jodice *et al.*, 2002 | *Rissa tridactyla* | seabird | chick mass | mean chick mass: 1998 (males & females) | 2.255 | 0.106 | 62 |
| 10 | Komdeur, 1996 | *Copsychus sechellarum* | small passerine | chick mass | mean fledging mass: 1990 (age 17 days old) | 4.002 | 1.201 | 10 |
| 11 | Martinez-Padilla *et al*., 2006 | *Falco tinnunculus* | bird of prey | chick mass | mean nestling mass: 2002 | -0.133 | 0.122 | 33 |
| 12 | Mock *et al*., 1987 | *Ardea alba* | wetland bird | chick mass | mean brood mass: 1985 (age 10 days old) | 1.215 | 0.047 | 100 |
| 13 | Nagy & Holmes, 1995 | *Dendroica caerulescens* | small passerine | chick mass | mean fledgling mass: 2000 (first nests) | 0.000 | 0.243 | 17 |
| 14 | Reynolds *et al*., 2003b | *Aphelocoma coerulescens* | corvid | chick mass | mean chick mass: 2001-2002 (age 11 days old) | -0.034 | 0.083 | 55 |
| 15 | Richner, 1992 | *Corvus corone* | corvid | chick mass | mean fledging mass: 1986-1989 (males & females) | 1.052 | 0.116 | 45 |
| 16 | Ritz *et al*., 2005 | *Catharacta maccormicki* | seabird | chick mass | mean k-value (inflexion point of the growth curve): 2001 | 0.000 | 0.074 | 54 |
| 17 | Sanz & Moreno, 1995 | *Ficedula hypoleuca* | small passerine | chick mass | mean fledgling mass: 1993 | -0.237 | 0.235 | 18 |
| 18 | Scheuerlein & Gwinner, 2006 | *Saxicola torquatus* | small passerine | chick mass | mean chick mass: 1995-1996 (age 2-7 days old) | 0.002 | 0.167 | 25 |
| 19 | Verboven *et al.*, 2001 | *Parus major* | small passerine | chick mass | mean fledgling mass: 1987-1990 | 0.628 | 0.037 | 161 |
| 20 | Verhulst, 1994 | *Ficedula hypoleuca* | small passerine | chick mass | mean nestling mass: 1987 | 0.165 | 0.151 | 27 |
| 21 | YomTov, 1974 | *Corvus corone* | corvid | chick mass | mean fledgling mass: 1972 (all chicks) | 2.329 | 0.396 | 17 |
| 1 | Arcese & Smith, 1988 | *Melospiza melodia* | small passerine | egg size | mean egg mass: 1985 | 0.159 | 0.096 | 49 |
| 2 | Arnold, 1992 | *Xanthocephalus xanthocephalus* | small passerine | egg size | mean egg volume: 1989 | -0.032 | 0.026 | 153 |
| 3 | Arnold, 1994 | *Fulica americana* | wetland bird | egg size | mean egg size: 1987-1989/1991 | 0.022 | 0.007 | 548 |
| 4 | Carlson, 1989 | *Lanius collurio* | small passerine | egg size | mean egg mass: 1984/1986 | 0.072 | 0.182 | 22 |
| 5 | Clifford & Anderson, 2001 | *Sula granti* | seabird | egg size | mean egg volume: 1996-1997 (first & second eggs) | 0.304 | 0.055 | 76 |
| 6 | De Neve *et al*., 2004 | *Pica pica* | corvid | egg size | mean egg size: 2000 | 0.489 | 0.037 | 155 |
| 7 | Eikenaar *et al*., 2003 | *Acrocephalus australis* | small passerine | egg size | mean egg volume: 2000-2001 | 0.186 | 0.079 | 68 |
| 8 | Hill, 1988 | *Fulica americana* | wetland bird | egg size | mean egg mass: 1982 | 5.429 | 2.688 | 7 |
| 9 | Hochachka & Boag, 1986 | *Pica pica* | corvid | egg size | mean egg volume: 1984-1985 | 0.327 | 0.080 | 53 |
| 10 | Horsfall, 1984 | *Fulica atra* | wetland bird | egg size | mean egg mass: 1979 (all eggs) | 0.311 | 0.137 | 30 |
| 11 | Högstetd, 1981 | *Pica pica* | small passerine | egg size | mean egg mass: 1974-1975 | 0.745 | 0.110 | 39 |
| 12 | Karell *et al*., 2008 | *Strix uralensis* | bird of prey | egg size | mean egg volume: 2004-2006 | 3.532 | 0.293 | 35 |
| 13 | Mackintosh & Briskie, 2005 | *Petroica australis* | small passerine | egg size | mean egg volume: 2001-2002 | 0.026 | 0.114 | 36 |
| 14 | Martinez-Padilla *et al*., 2006 | *Falco tinnunculus* | bird of prey | egg size | mean egg mass: 2002 | -0.120 | 0.122 | 33 |
| 15 | Millon *et al*., 2008 | *Circus pygargus* | bird of prey | egg size | mean egg volume: all years (sites D/R) | 0.094 | 0.039 | 156 |
| 16 | Nilsson & Svensson, 1993 | *Cyanistes caeruleus* | small passerine | egg size | mean egg mass: 1990 (prelaying supplementation) | 0.115 | 0.060 | 79 |
| 17 | Pihlaja *et al.*, 2006 | *Pica pica* | corvid | egg size | mean egg weight: 2002 | 0.686 | 0.224 | 19 |
| 18 | Poole, 1985 | *Pandion haliaetus* | bird of prey | egg size | mean egg size: 1980 | -0.475 | 0.514 | 8 |
| 19 | Ramsay & Houston, 1998 | *Cyanistes caeruleus* | small passerine | egg size | mean egg mass: 1995 (high protein treatment) | 0.326 | 0.101 | 40 |
| 20 | Reynolds *et al*., 2003 | *Aphelocoma coerulescens* | corvid | egg size | mean egg mass: 2000-2001 (first eggs - high fat/high protein) | 0.364 | 0.067 | 76 |
| 21 | Sanz & Moreno, 1995 | *Ficedula hypoleuca* | small passerine | egg size | mean egg mass: 1993 | -0.122 | 0.134 | 30 |
| 22 | Soler & Soler, 1996 | *Corvus monedula* | corvid | egg size | mean egg size: 1983 | 0.326 | 0.165 | 26 |
| 23 | Spottiswoode, 2009 | *Philetairus socius* | small passerine | egg size | mean egg mass: 2004 | 0.301 | 0.043 | 96 |
| 24 | Styrsky *et al*., 2000 | *Troglodytes aedon* | small passerine | egg size | mean egg mass: 1998 | 0.714 | 0.116 | 43 |
| 25 | Wiebe & Bortolotti, 1994 | *Falco sparverius* | bird of prey | egg size | mean egg volume: 1990-1991 | 0.842 | 0.057 | 76 |
| 26 | Zanette *et al*., 2009 | *Melospiza melodia* | small passerine | egg size | mean egg mass: 2000-2002 | -0.408 | 0.021 | 197 |
| 1 | Bolton *et al*., 1992 | *Larus fuscus* | seabird | hatching success | mean hatching success: 1990 (egg fed) | 0.322 | 0.069 | 59 |
| 2 | Castro *et al*., 2003 | *Notiomystis cincta* | small passerine | hatching success | mean hatching success: 1995/1997 | 0.223 | 0.115 | 35 |
| 3 | Clamens & Insenmann, 1989 | *Cyanistes caeruleus* | small passerine | hatching success | mean hatching success: 1986-1987 | -0.229 | 0.078 | 59 |
| 4 | Clamens & Insenmann, 1989 | *Parus major* | small passerine | hatching success | mean hatching success: 1986-1987 | -0.334 | 0.132 | 31 |
| 5 | Eikenaar *et al*., 2003 | *Acrocephalus australis* | small passerine | hatching success | mean hatching success: 2000-2001 | 0.313 | 0.109 | 53 |
| 6 | Gill & Hatch, 2002 | *Rissa tridactyla* | seabird | hatching success | mean hatching success: 1996-1997 (fed all seasons) | 0.746 | 0.030 | 168 |
| 7 | Harrison *et al.*, 2010 | *Cyanistes caeruleus* | small passerine | hatching success | mean hatching success: 2006-2008 | -0.274 | 0.013 | 352 |
| 8 | Harrison *et al*., 2010 | *Parus major* | small passerine | hatching success | mean hatching success: 2006-2008 | 0.051 | 0.019 | 242 |
| 9 | Hoodless *et al.*, 1999 | *Phasianus colchicus* | other | hatching success | mean hatching success: 1994-1995 | 1.657 | 0.539 | 13 |
| 10 | Komdeur, 1996 | *Copsychus sechellarum* | small passerine | hatching success | mean hatching success: 1990 | 0.501 | 0.413 | 10 |
| 11 | Mackintosh & Briskie, 2005 | *Petroica australis* | small passerine | hatching success | mean hatching success: 2001-2002 | 1.331 | 0.110 | 45 |
| 12 | Millon *et al*., 2008 | *Circus pygargus* | bird of prey | hatching success | mean hatching success: all years (sites D/R) | 0.000 | 0.060 | 122 |
| 13 | Nilsson, 1994 | *Cyanistes caeruleus* | small passerine | hatching success | mean hatching success: 1991 | -0.184 | 0.106 | 56 |
| 14 | Pihlaja *et al*., 2006 | *Pica pica* | corvid | hatching success | mean hatching success: 2002 | 0.071 | 0.211 | 19 |
| 15 | Poole, 1985 | *Pandion haliaetus* | bird of prey | hatching success | mean hatching success: 1980 | -0.988 | 0.561 | 8 |
| 16 | Sanz & Moreno, 1995 | *Ficedula hypoleuca* | small passerine | hatching success | mean hatching success: 1993 | -0.598 | 0.149 | 28 |
| 17 | Soler & Soler, 1996 | *Corvus monedula* | corvid | hatching success | mean hatching success: 1983 | 0.074 | 0.133 | 31 |
| 18 | Wellicome, 2005 | *Athene cunicularia* | bird of prey | hatching success | mean hatching success: 1992-1993-1996 | 0.141 | 0.044 | 92 |

**References**

Arcese P, Smith JNM: **Effects of population density and supplemental food on reproduction in song sparrows**. *J Anim Ecol* 1988, **57**:119–136.

Arnold TW: **Variation in laying date, clutch size, egg size, and egg composition of yellow-headed blackbirds (*Xanthocephalus xanthocephalus*): a supplemental feeding experiment**. *Can J Zool* 1992, **70**:1904–1911.

Arnold TW: **Effect of supplemental food on egg production in American coots**. *Auk* 1994, **111**:337–350.

Bolton M, Houston DC, Monaghan P: **Nutritional constraints on egg formation in the lesser black-backed gull: an experimental study**. *J Anim Ecol* 1992, **61**:521–532.

Carlson A: **Courtship feeding and clutch size in Red-Backed Shrikes (*Lanius collurio*)**. *Am Nat* 1989, **133**:454–457.

Castro I, Brunton DH, Mason KM, Ebert B, Griffiths R: **Life history traits and food supplementation affect productivity in a translocated population of the endangered Hihi (Stitchbird, *Notiomystis cincta*)**. *Biol Conserv* 2003, **114**:271–280.

Clamens A, Isenmann P: **Effect of supplemental food on the breeding of Blue and Great Tits in Mediterranean habitats**. *Ornis Scand* 1989, **20**:36–42.

Clifford LD, Anderson DJ: **Food limitation explains most clutch size variation in the Nazca booby**. *J Anim Ecol* 2001, **70**:539–545.

Clinchy M, Zanette L, Boonstra R, Wingfield JC, Smith JNM: **Balancing food and predator pressure induces chronic stress in songbirds.** *Proc Biol Sci* 2004, **271**:2473–2479.

Davies NB, Lundberg A: **The influence of food on time budget and timing of breeding of the Dunnock *Prunella modularis***. *Ibis* 1985, **127**:100–110.

De Neve L, Soler JJ, Soler M, Martín-Vivaldi M, Martínez JG: **Effects of a food supplementation experiment on reproductive investment and a post-mating sexually selected trait in magpies *Pica pica***. *J Avian Biol* 2004, **35**:246–251.

Dhindsa MS, Boag DA: **The effect of food supplementation on the reproductive success of Black-billed Magpies *Pica pica***. *Ibis* 1990, **132**:595–602.

Eikenaar C, Berg ML, Komdeur J: **Experimental evidence for the influence of food availability on incubation attendance and hatching asynchrony in the Australian reed warbler *Acrocephalus australis***. *J Avian Biol* 2003, **34**:419–427.

Ewald PW, Rohwer S: **Effects of supplemental feeding on timing of breeding, clutch-size and polygyny in red-winged blackbirds *Agelaius phoeniceus***. *J Anim Ecol* 1982, **51**:429–450.

Garcia PFJ, Merkle MS, Barclay RMR: **Energy allocation to reproduction and maintenance in mountain bluebirds (*Sialia currucoides*): a food supplementation experiment**. *Can J Zool* 1993, **71**:2352–2357.

Gill VA, Hatch SA: **Components of productivity in black-legged kittiwakes *Rissa tridactyla*: response to supplemental feeding**. *J Avian Biol* 2002, **33**:113–126.

Harris PM: **Supplementary feeding of young puffind, Fratercula arctica**. *J Anim Ecol* 1978, **47**:55–23.

Harrison TJE, Smith JA, Martin GR, Chamberlain DE, Bearhop S, Robb GN, Reynolds SJ: **Does food supplementation really enhance productivity of breeding birds?** *Oecologia* 2010, **164**:311–20.

Hill WL: **The effect of food abundance on the reproductive patterns of coots**. *Condor* 1988, **90**:324–331.

Hillström L: **Body mass reductionin during reproduction in the Pied Flycatcher *Ficedula hypoleuca*: physiological stress or adaptation for lowered costs of locomotion?** *Funct Ecol* 1995, **9**:807–817.

Hipkiss T, Hörnfeldt B, Eklund U, Berlin S: **Year-dependent sex-biased mortality in supplementary-fed Tengmalm’s owl nestlings**. *J Anim Ecol* 2002, **71**:693–699.

Hochachka WM, Boag A: **Food shortage for breeding Black-billed Magpies (*Pica pica*): an experiment using supplemental food**. *Can J Zool* 1987, **65**:1270–1274.

Hoodless AN, Draycott R a. H, Ludiman MN, Robertson PA: **Effects of supplementary feeding on territoriality, breeding success and survival of pheasants**. *J Appl Ecol* 1999, **36**:147–156.

Horsfall JA: **Food supply and egg mass variation in the European coot**. *Ecology* 1984, **65**:89–95.

Hogstedt G: **Effect of additional food on reproductive success in the Magpie (Pica pica)**. *J Anim Ecol* 1981, **50**:219–229.

Jodice PGR, Roby DD, Hatch SA, Gill VA, Lanctot RB, Visser GH: **Does food availability affect energy expenditure rates of nesting seabirds? A supplemental-feeding experiment with Black-legged Kittiwakes (*Rissa tridactyla*)**. *Can J Zool* 2002, **222**:214–222.

Karell P, Kontiainen P, Pietiinen H, Siitari H, Brommer JE: **Maternal effects on offspring Igs and egg size in relation to natural and experimentally improved food supply**. *Funct Ecol* 2008, **22**:682–690.

Komdeur J: **Breeding of the Seychelles Magpie Robin Copsychus sechellarum and implications for its conservation**. *Ibis* 1996, **138**:485–498.

Mackintosh M, Briskie J: **High levels of hatching failure in an insular population of the South Island robin: a consequence of food limitation?** *Biol Conserv* 2005, **122**:409–416.

Martínez-Padilla J: **Prelaying maternal condition modifies the association between egg mass and T cell-mediated immunity in kestrels**. *Behav Ecol Sociobiol* 2006, **60**:510–515.

Millon A, Arroyo BE, Bretagnolle V: **Variable but predictable prey availability affects predator breeding success: natural versus experimental evidence**. *J Zool* 2008, **275**:349–358.

Mock DW, Lamey TC, Ploger BJ: **Proximate and ultimate roles of food amount in regulating egret sibling aggression**. *Ecology* 1987, **68**:1760–1772.

Nagy LR, Holmes RT: **Food limits annual fecundity of a migratory songbird: an experimental study**. *Ecology* 2005, **86**:675–681.Nilsson J-Å, Svensson E: **Energy constraints and ultimate decisions during egg-laying in the blue tit**. *Ecology* 1993, **74**:244–251.

Nilsson J-Å: **Energetic bottle-necks during breeding and the reproductive cost of being too early**. *J Anim Ecol* 1994, **63**:200–208.

Pihlaja M, Siitari H, Alatalo RV: **Maternal antibodies in a wild altricial bird: effects on offspring immunity, growth and survival.** *J Anim Ecol* 2006, **75**:1154–1164.

Poole A: **Courtship feeding and osprey reproduction**. *Auk* 1985, **102**:479–492.

Ramsay SL, Houston DC: **The effect of dietary amino acid composition on egg production in blue tits**. *Proc R Soc B Biol Sci* 1998, **265**:1401–1405.

Reynolds SJ, Schoech SJ, Bowman R: **Nutritional quality of prebreeding diet influences breeding performance of the Florida scrub-jay.** *Oecologia* 2003a, **134**:308–316.

Reynolds SJ, Schoech SJ, Bowman R: **Diet quality during pre-laying and nestling periods influences growth and survival of Florida scrub-jay (*Aphelocoma coerulescens*) chicks**. *J Zool* 2003b, **261**:217–226.

Richner H: **The effect of extra food on fitness in breeding carrion crows**. *Ecology* 1992, **73**:330–335.

Ritz MS, Hahn S, Peter H-U: **Factors affecting chick growth in the South Polar Skua (*Catharacta maccormicki*): food supply, weather and hatching date**. *Polar Biol* 2005, **29**:53–60.

Sanz JJ, Moreno J: **Experimentally induced clutch size enlargements affect reproductive success in the Pied Flycatcher**. *Oecologia* 1995, **103**:358–364.

Scheuerlein A, Gwinner E: **Reduced nestling growth of East African Stonechats Saxicola torquata axillaris in the presence of a predator**. *Ibis* 2006, **148**:468–476.Soler M, Soler JJ: **Effects of experimental food provisioning on reproduction in the jackdaw *Corvus monedula*, a semi-colonial species**. *Ibis*  1996, **138**:377–383.

Spottiswoode CN: **Fine-scale life-history variation in sociable weavers in relation to colony size.** *J Anim Ecol* 2009, **78**:504–12.

Styrsky JD, Dobbs RC, Thompson CF: **Food-supplementation does not override the effect of egg mass on fitness-related traits of nestling house wrens**. *J Anim Ecol* 2000, **69**:690–702.

Wellicome TI: **Hatching asynchrony in Burrowing Owls is influenced by clutch size and hatching success but not by food.** *Oecologia* 2005, **142**:326–34.

Verboven N, Tinbergen JM, Verhulst S: **Food, reproductive success and multiple breeding in the great tit *Parus major****.* *Ardea* 2001, **89**:387–406.

Verhulst S: **Supplementary food in the nestling phase affects reproductive success in pied flycatchers (*Ficedula hypoleuca*)**. *Auk* 1994, **111**:714–716.

Wiebe KL, Bortolotti GR: **Food supply and hatching spans of birds: energy constraints or facultative manipulation**. *Ecology* 1994, **75**:813–823.

Yom-tov Y: **The effect of food and predation on breeding density and success, clutch size and laying date of the crow (*Corvus corone* L.)**. *J Anim Ecol* 1974, **43**:479–498.

Zanette L, Clinchy M, Smith JNM: **Food and predators affect egg production in song sparrows.** *Ecology* 2006, **87**:2459–67.

**3e**. List of all the publications included in the meta-analyses testing the effect the background level of food abundance on the outcomes of supplementation experiments, sorted by reference.

|  | **Reference** | **Species** | **BirdType** | **FoodLevel** | **Parameter** | **Response** | ***d*** | **Var(*d*)** | **Nt** |
| --- | --- | --- | --- | --- | --- | --- | --- | --- | --- |
| 1 | Bolton *et al*. 1992 | *Larus fuscus* | seabird | lower | CS | mean clutch size: 1989 (fish supplemented) | 0.584 | 0.080 | 55 |
| 2 | Bolton *et al*. 1992 | *Larus fuscus* | seabird | higher | CS | mean clutch size: 1990 (fish supplemented) | 0.016 | 0.069 | 59 |
| 3 | Bourgault *et al.* 2009 | *Parus caerulus* | small passerine | lower | LD | mean laying date: 2006-2007 (evergreen Muro) | 1.194 | 0.072 | 66 |
| 4 | Bourgault *et al.* 2009 | *Parus caerulus* | small passerine | high | LD | mean laying date: 2006-2007 (deciduous Muro/Pioggiola/Pirio) | -0.234 | 0.017 | 236 |
| 5 | von Brömssen & Jansson, 1990 | *Parus montanus* | small passerine | higher | CS | mean clutch size: 1976 | 0.042 | 0.220 | 20 |
| 6 | von Brömssen & Jansson, 1990 | *Parus montanus* | small passerine | lower | CS | mean clutch size: 1977 | 0.271 | 0.224 | 24 |
| 7 | von Brömssen & Jansson, 1990 | *Parus cristatus* | small passerine | higher | CS | mean clutch size: 1976 | -0.068 | 0.389 | 21 |
| 8 | von Brömssen & Jansson, 1990 | *Parus cristatus* | small passerine | lower | CS | mean clutch size: 1977 | 0.393 | 0.288 | 17 |
| 9 | Byholm & Kekkonen, 2008 | *Accipiter gentilis* | bird of prey | higher | BS | mean breeding success (forest) | -0.315 | 0.124 | 35 |
| 10 | Byholm & Kekkonen, 2008 | *Accipiter gentilis* | bird of prey | lower | BS | mean breeding success (bog) | 0.526 | 0.080 | 52 |
| 11 | Dijkstra *et al*., 1982 | *Falco tinnunculus* | bird of prey | lower | LD | mean laying date: 1978-1979 | 2.104 | 0.388 | 17 |
| 12 | Dijkstra *et al*., 1982 | *Falco tinnunculus* | bird of prey | higher | LD | mean laying date: 1980 | 0.169 | 0.390 | 21 |
| 13 | Dijkstra *et al*., 1982 | *Falco tinnunculus* | bird of prey | lower | CS | mean clutch size: 1978-1979 | 1.231 | 0.338 | 16 |
| 14 | Dijkstra *et al*., 1982 | *Falco tinnunculus* | bird of prey | higher | CS | mean clutch size: 1980 | 0.563 | 0.567 | 19 |
| 15 | Gill & Hatch, 2002 | *Rissa tridactyla* | seabird | higher | CS | mean clutch size: 1996 (fed all seasons) | 0.080 | 0.057 | 84 |
| 16 | Gill & Hatch, 2002 | *Rissa tridactyla* | seabird | lower | CS | mean clutch size: 1997 (fed all seasons) | 0.380 | 0.058 | 84 |
| 17 | Gill & Hatch, 2002 | *Rissa tridactyla* | seabird | higher | BS | mean breeding success: 1996 (fed all seasons) | 0.913 | 0.075 | 65 |
| 18 | Gill & Hatch, 2002 | *Rissa tridactyla* | seabird | lower | BS | mean breeding success: 1997 (fed all seasons) | 0.839 | 0.077 | 63 |
| 19 | Granbom & Smith, 2006 | *Sturnus vulgaris* | small passerine | lower | BS | mean breeding success (lowest quality habitat) | 0.470 | 0.072 | 57 |
| 20 | Granbom & Smith, 2006 | *Sturnus vulgaris* | small passerine | higher | BS | mean breeding success (highest quality habitat) | 0.416 | 0.098 | 42 |
| 21 | Hiom *et al*., 1991 | *Larus fuscus* | seabird | lower | CS | mean clutch size (Skomer) | 0.021 | 0.067 | 65 |
| 22 | Hiom et al., 1991 | *Larus fuscus* | seabird | higher | CS | mean clutch size (FlatHolm) | 0.026 | 0.077 | 55 |
| 23 | Korpimäki, 1989 | *Aegolius funereus* | bird of prey | higher | LD | mean laying date: 1986 | 1.111 | 0.191 | 25 |
| 24 | Korpimäki, 1989 | *Aegolius funereus* | bird of prey | higher | CS | mean clutch size: 1986 | -1.009 | 0.187 | 25 |
| 25 | Korpimäki, 1989 | *Aegolius funereus* | bird of prey | higher | BS | mean breeding success | 0.000 | 0.167 | 25 |
| 26 | Korpimäki & Wiehn, 1998 | *Falco tinnunculus* | bird of prey | lower | LD | mean laying date: 1986 | 0.127 | 0.225 | 18 |
| 27 | Korpimäki & Wiehn, 1998 | *Falco tinnunculus* | bird of prey | higher | LD | mean laying date: 1988 | -0.031 | 0.096 | 22 |
| 28 | Korpimäki & Wiehn, 1998 | *Falco tinnunculus* | bird of prey | lower | CS | mean clutch size: 1986 | 1.036 | 0.255 | 18 |
| 29 | Korpimäki & Wiehn, 1998 | *Falco tinnunculus* | bird of prey | higher | CS | mean clutch size: 1988 | 1.145 | 0.111 | 22 |
| 30 | Hörnfeldt et al., 2000 | *Aegolius funereus* | bird of prey | higher | BS | mean breeding success: 1998 | -0.661 | 0.177 | 24 |
| 31 | Millon *et al*., 2008 | *Circus pygargus* | bird of prey | lower | LD | mean laying date: low rodent years | 0.661 | 0.104 | 57 |
| 32 | Millon *et al*., 2008 | *Circus pygargus* | bird of prey | higher | LD | mean laying date: high rodent years | -0.045 | 0.074 | 84 |
| 33 | Millon *et al*., 2008 | *Circus pygargus* | bird of prey | lower | CS | mean clutch size: low rodent years | 0.844 | 0.106 | 57 |
| 34 | Millon *et al*., 2008 | *Circus pygargus* | bird of prey | higher | CS | mean clutch size: high rodent years | 0.225 | 0.074 | 84 |
| 35 | Millon *et al*., 2008 | *Circus pygargus* | bird of prey | lower | BS | mean fledging success: low rodent years | 0.101 | 0.132 | 58 |
| 36 | Millon *et al*., 2008 | *Circus pygargus* | bird of prey | higher | BS | mean fledging success: high rodent years | -0.173 | 0.110 | 64 |
| 37 | Meijer *et al*., 1988 | *Falco tinnunculus* | bird of prey | higher | LD | mean laying date: high rodent years (early feeding) | -0.384 | 0.259 | 23 |
| 38 | Meijer *et al*., 1988 | *Falco tinnunculus* | bird of prey | lower | LD | mean laying date: low rodent years (early feeding) | 1.634 | 0.232 | 26 |
| 39 | Meijer *et al*., 1988 | *Falco tinnunculus* | bird of prey | higher | CS | mean clutch size: high rodent years (early feeding) | -0.143 | 0.259 | 22 |
| 40 | Meijer *et al*., 1988 | *Falco tinnunculus* | bird of prey | lower | CS | mean clutch size: low rodent years (early feeding) | 0.584 | 0.233 | 23 |
| 41 | Nagy & Holmes, 2005 | *Dendroica caerulescens* | small passerine | higher | BS | mean breeding success: 2000 | 0.470 | 0.249 | 17 |
| 42 | Nagy & Holmes, 2005 | *Dendroica caerulescens* | small passerine | lower | BS | mean breeding success: 2001 | -0.579 | 0.220 | 19 |
| 43 | Newton & Marquiss, 1981 | *Accipiter nisus* | bird of prey | lower | LD | mean laying date: 1974-1975/1976-1978 | 0.927 | 0.091 | 113 |
| 44 | Newton & Marquiss, 1981 | *Accipiter nisus* | bird of prey | lower | CS | mean clutch size: 1974-1975/1976-1978 | 1.122 | 0.093 | 113 |
| 45 | Preston & Rotenberry, 2006 | *Chamaea fasciata* | small passerine | higher | BS | mean breeding success: 2001 | 0.595 | 0.102 | 41 |
| 46 | Preston & Rotenberry, 2006 | *Chamaea fasciata* | small passerine | lower | BS | mean breeding success: 2002 | 1.031 | 0.216 | 26 |
| 47 | Reynolds *et al*., 2003 | *Aphelocoma coerulescens* | corvid | lower | BS | mean breeding success: 2001 | 3.225 | 0.350 | 28 |
| 48 | Reynolds *et al*., 2003 | *Aphelocoma coerulescens* | corvid | higher | BS | mean breeding success: 2002 | -0.191 | 0.167 | 27 |
| 49 | Wiehn & Korpimäki, 1997/Wiehn *et al.* 2000 | *Falco tinnunculus* | bird of prey | lower | BS | mean breeding success: 1993 | 0.265 | 0.142 | 31 |
| 50 | Wiehn & Korpimäki, 1997/Wiehn *et al.* 2000 | *Falco tinnunculus* | bird of prey | higher | BS | mean breeding success: 1992/1995 | 0.378 | 0.059 | 78 |
| 51 | Wiehn & Korpimäki, 1997/Wiehn *et al.* 2000 | *Falco tinnunculus* | bird of prey | lower | BS | mean breeding success: 1997 | 0.770 | 0.105 | 41 |

**References**

Bolton M, Houston DC, Monaghan P: **Nutritional constraints on egg formation in the lesser black-backed gull: an experimental study**. *J Anim Ecol* 1992, **61**:521–532.

Bourgault P, Perret P, Lambrechts MM: **Food supplementation in distinct Corsican oak habitats and the timing of egg laying by Blue Tits**. *J F Ornithol* 2009, **80**:127–134.

von Brömssen A, Jansson C: **Effects of food addition to Willow Tit *Parus montanu*s and Crested Tit *P. cristatus* at the time of breeding**. *Ornis Scand* 1980, **11**:173–178.

Byholm P, Kekkonen M: **Food regulates reproduction differently in different habitats: experimental evidence in the Goshawk.** *Ecology* 2008, **89**:1696–702.

Dijkstra C, Vuursteen L, Daan S, Masman D: **Clutch size and laying date in the kestrel *Falco tinnunculus*: effect of supplementary food**. *Ibis* 1982, **124**:210–213.

Gill VA, Hatch SA: **Components of productivity in black-legged kittiwakes *Rissa tridactyla*: response to supplemental feeding**. *J Avian Biol* 2002, **33**:113–126.

Granbom M, Smith HG: **Food limitation during breeding in a heterogeneous landscape**. *Auk* 2006, **123**:97.

Hiom L, Bolton M, Monaghan P, Worrall D: **Experimental evidence for food limitation of egg production in gulls**. *Ornis Scand* 1991, **22**:94–97.

Hörnfeldt B, Hipkiss T, Fridolfsson A-K, Eklund U, Ellegren H: **Sex ratio and fledging success of supplementary-fed Tengmalm´s owl broods**. *Mol Ecol* 2000, **9**:187–192.

Korpimäki E: **Breeding performance of Tengmalm’s Owl Aegolius funereus: effects of supplementary feeding in a peak vole year**. *Ibis* 1989, **131**:51–56.

Meijer T, Daan S, Dijkstra C: **Female condition and reproduction: effects of food manipulation in free-living and captive kestrels**. *Ardea* 1988, **76**:141–154.

Millon A, Arroyo BE, Bretagnolle V: **Variable but predictable prey availability affects predator breeding success: natural versus experimental evidence**. *J Zool* 2008, **275**:349–358.

Nagy LR, Holmes RT: **Food limits annual fecundity of a migratory songbird: an experimental study**. *Ecology* 2005, **86**:675–681.

Newton I, Marquiss M: **Effect of additional food on laying dates and clutch sizes of Sparrowhawks**. *Ornis Scand* 1981, **12**:224–229.

Preston KL, Rotenberry JT: **Independent effects of food and predator-mediated processes on annual fecundity in a songbird.** *Ecology* 2006, **87**:160–168.

Reynolds SJ, Schoech SJ, Bowman R: **Diet quality during pre-laying and nestling periods influences growth and survival of Florida scrub-jay (*Aphelocoma coerulescens*) chicks**. *J Zool* 2003, **261**:217–226.

Wiehn J, Korpimäki E: **Food limitation on brood size: experimental evidence in the Eurasian kestrel**. *Ecology* 1997, **78**:2043–2050.

Wiehn J, Ilmonen P, Korpimäki E, Pahakala M, Wiebe KL: **Hatching asynchrony in the Eurasian kestrel *Falco tinnunculus*: an experimental test of the brood reduction hypothesis**. *J Anim Ecol* 2000, **69**:85–95.
