# Supplementary material for: Reproductive responses of birds to experimental food supplementation: a meta-analysis
Source: Front Zool. 2014 Oct 31;11:80. doi: 10.1186/s12983-014-0080-y (PMC4222371; doi:10.1186/s12983-014-0080-y)
Supplement: Additional file 4: — Phylogenetic trees used in the phylogenetic meta-analyses. [file 12983_2014_80_MOESM4_ESM.docx]

**Additional file 4. Phylogenetic trees used in the phylogenetic meta-analyses**

Phylogenetic trees were constructed for each of the eight datasets. Trees were extracted from birdtree.org based on the Ericson class-wide distribution. We then used the Maximum Clade Credibility option in TreeAnnotator (v1.8.0) to summarize the information from a sample of 2000 to 5000 possible trees onto a single target tree. Note that *Parus caeruleus* refers to *Cyanistes caeruleus*, *Parus palustris* to *Poecile palustris*, *Parus montanus* to *Poecile montanus*, and *Parus cristatus* to *Lophophanes cristatus*.

*Phylogenetic tree for the bird species included in the brood size dataset*

**

*Phylogenetic tree for the bird species included in the laying date dataset*

*Phylogenetic tree for the bird species included in the clutch size dataset*

*Phylogenetic tree for the bird species included in the breeding success dataset*

*Phylogenetic tree for the bird species included in the egg size dataset*

*Phylogenetic tree for the bird species included in the hatching success dataset*

*Phylogenetic tree for the bird species included in the chick body mass dataset*

*Phylogenetic tree for the bird species included in the background food level dataset*
