# Supplementary material for: Reproductive responses of birds to experimental food supplementation: a meta-analysis
Source: Front Zool. 2014 Oct 31;11:80. doi: 10.1186/s12983-014-0080-y (PMC4222371; doi:10.1186/s12983-014-0080-y)

**Additional file 5. Examination of publication bias**

We examined the possibility of publication bias in our datasets by generating funnel plots and QQ-plots based on the null random models. Funnel plots show the residuals of the models on the horizontal axis against their corresponding standard errors on the vertical axis. Asymmetry in a funnel plot would indicate a significant number of studies (probably unpublished with non-significant findings) being omitted from the analyses. We also computed the Orwin fail-safe number for each dataset, which calculates the number of studies averaging null results that would have to be added to the given set of observed outcomes to reduce the (unweighted) average effect size to a target (unweighted) average effect size

The regression tests for funnel plot asymmetry on the null laying date (z = 1.80, P = 0.06; Fig. A1a), clutch size (z = -0.60, P = 0.55; Fig. A2a) and breeding success (z = 1.18, P = 0.24; Fig. A3a) models, the examination of the corresponding Q-Q plots (Fig. 5A.a; Fig. 5A.b, Fig. 5A.c) and fail-safe numbers indicate no publication bias in any of the datasets.

**Figure 5A.a.** Funnel plot of the null laying date model

Note: Orwin fail-safe number is 41.

**Figure 5A.b.** QQ-plot of the null laying date model

**
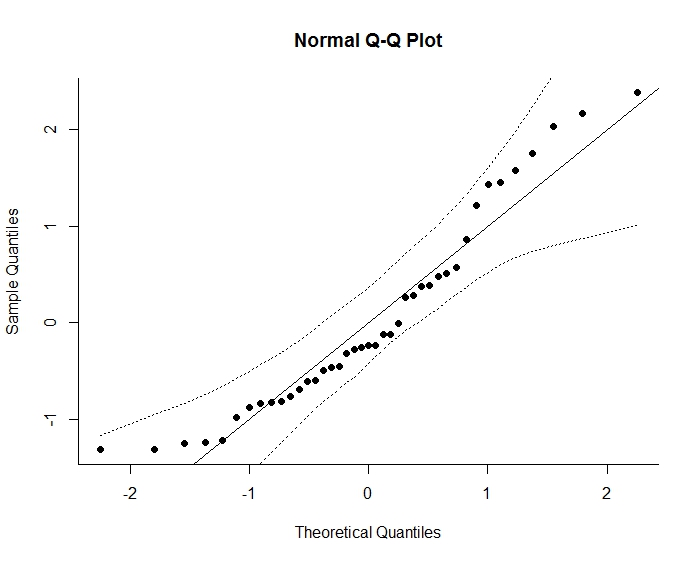
**

**Figure 5B.a**. Funnel plot for the null clutch size model

Note: Orwin fail-safe number is 46.

**Figure 5B.b.** QQ-plot of the null clutch size model


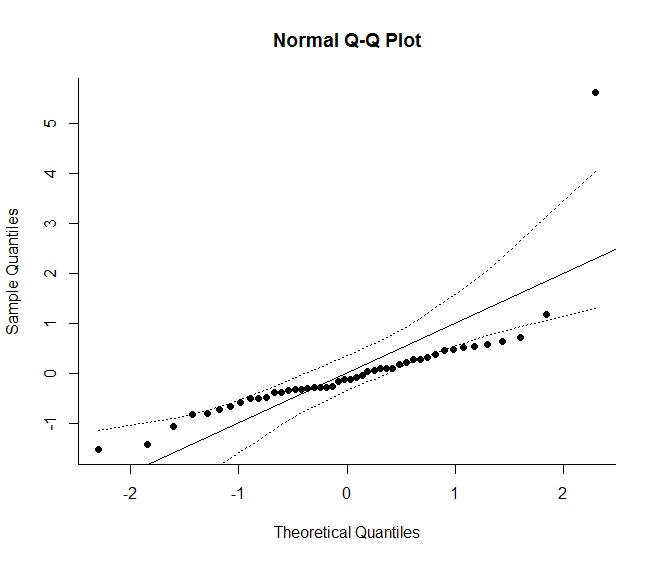


**Figure 5C.a**. Funnel plot for the null breeding success model

Note: Orwin fail-safe number is 38.

**Figure 5C.b.** QQ-plot of the null breeding success model


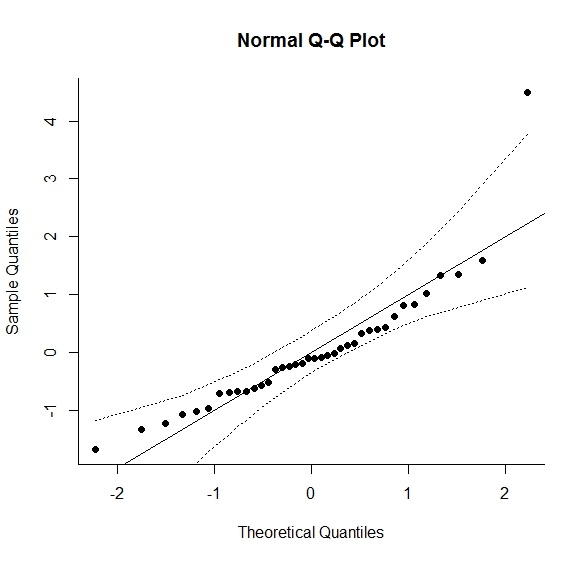

Supplement: Additional file 5: — Examination of publication bias. [file 12983_2014_80_MOESM5_ESM.docx]
